# Interactions Between Climate Mean and Variability Drive Future Agroecosystem Vulnerability

**DOI:** 10.1111/gcb.70064

**Published:** 2025-02-07

**Authors:** Eva Sinha, Donghui Xu, Kendalynn A. Morris, Beth A. Drewniak, Ben Bond‐Lamberty

**Affiliations:** ^1^ Atmospheric, Climate, and Earth Sciences Division Pacific Northwest National Laboratory Richland Washington USA; ^2^ Joint Global Change Research Institute Pacific Northwest National Laboratory College Park Maryland USA; ^3^ Environmental Science Division Argonne National Laboratory Lemont Illinois USA

**Keywords:** agroecosystem, climate impact, nonlinear

## Abstract

Agriculture is crucial for global food supply and dominates the Earth's land surface. It is unknown, however, how slow but relentless changes in climate *mean* state, versus random extreme conditions arising from changing *variability*, will affect agroecosystems' carbon fluxes, energy fluxes, and crop production. We used an advanced weather generator to partition changes in mean climate state versus variability for both temperature and precipitation, producing forcing data to drive factorial‐design simulations of US Midwest agricultural regions in the Energy Exascale Earth System Model. We found that an increase in temperature mean lowers stored carbon, plant productivity, and crop yield, and tends to convert agroecosystems from a carbon sink to a source, as expected; it also can cause local to regional cooling in the earth system model through its effects on the Bowen Ratio. The combined effect of mean and variability changes on carbon fluxes and pools was nonlinear, that is, greater than each individual case. For instance, gross primary production reduces by 9%, 1%, and 13% due to change in mean temperature, change in temperature variability, and change in both temperature mean and variability, respectively. Overall, the scenario with change in both temperature and precipitation means leads to the largest reduction in carbon fluxes (−16% gross primary production), carbon pools (−35% vegetation carbon), and crop yields (−33% and −22% median reduction in yield for corn and soybean, respectively). By unambiguously parsing the effects of changing climate mean versus variability and quantifying their nonadditive impacts, this study lays a foundation for more robust understanding and prediction of agroecosystems' vulnerability to 21st‐century climate change.

## Graphical abstract

Agroecosystems are a global food supply source that impacts local and regional climates, but their vulnerability to changes in climate mean or increased variability remains poorly understood. In this manuscript, we used an advanced weather generator to partition changes in mean climate state versus variability and use that to drive factorial‐design simulations of U.S. Midwest agricultural regions in the Energy Exascale Earth System Model. We show that the effect of change in climate mean and variability on carbon fluxes and pools is nonlinear.

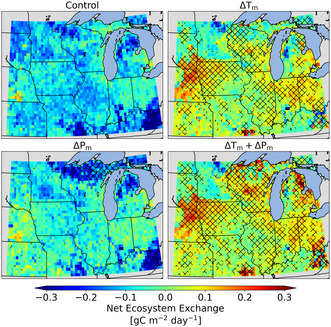

## 1 Introduction

Agriculture feeds the world's human population and has an enormous global footprint, with half of habitable land area used for food production (FAO 2024), livestock grazing, and other agricultural activities. Conversion of native vegetation to agroecosystems and various agricultural management activities releases large quantities of greenhouse gases to the atmosphere and impacts surface energy balances. Currently, agricultural activities produce more than 5 billion metric ton of ${\mathrm{CO}}_{2}$ equivalent annually (Tubiello 2019; Tubiello et al. 2021). Food production now accounts for 26%–34% of GHG emissions each year (Poore and Nemecek 2018; Crippa et al. 2021), while land use change and loss of soil organic carbon due to agricultural activities has contributed 214 Pg C to the atmosphere since 1850 (Zomer et al. 2017). These processes and interactions modify local to global climate.

Agroecosystems will clearly be affected by future changes in mean climate (Zhao et al. 2017) but are also vulnerable to extremes such as heat waves and droughts driven by an increase in climate variability. Such climate extremes have been projected to cause global declines in cereal production (Lesk, Rowhani, and Ramankutty 2016) and increase in CO_2_ emissions (An et al. 2022) while strongly affecting plant production (Thomey et al. 2011), carbon cycling (Rastetter et al. 2023), and plant mortality (Neumann et al. 2017). An example is the historical US drought in 2012 that resulted in widespread agricultural losses amounting to 30 billion in cost (Rippey 2015). Overall, it is estimated that historical droughts and heat waves have reduced national crop yields by up to 10% (Lesk, Rowhani, and Ramankutty 2016).

It is uncertain, however, whether change in climate mean or increased variability will have the most impact on food production and climate feedbacks, and to what degree these changes might interact with each other. This is made more difficult because state‐of‐the‐art Earth System Models (ESMs) robustly predict changes in climatological means but have more difficulty in predicting changes in variability (Lee et al. 2021). Additionally, ESM climate projections such as CMIP6 (Eyring et al. 2016) combine the impact of mean and variability changes in their standard simulations, making it challenging to understand the individual impacts and thus assess possible mitigation strategies tailored to different risks (Anderegg et al. 2020).

**FIGURE 1:**
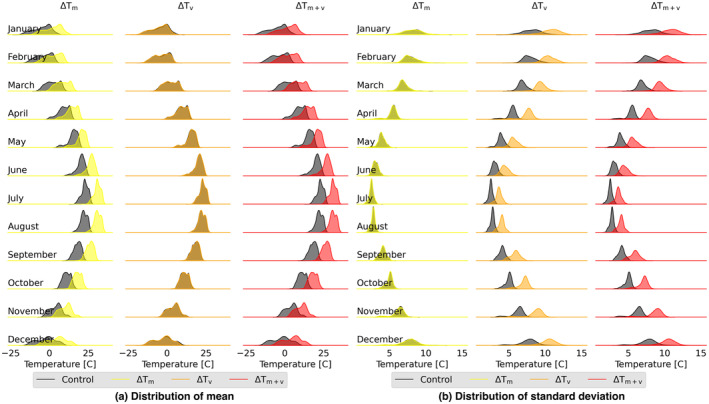
Modification of temperature forcing for the future sets of simulations: Distribution of mean (a) and standard deviation (b) of daily temperature (°C) for each grid cell. The control represents current conditions; $\Delta T_{m}$ accounts for change in temperature mean; $\Delta T_{v}$ accounts for change in temperature variability; and $\Delta T_{m+v}$ accounts for change in both temperature mean and variability. The changes in temperature mean and variability were based on factors‐of‐change derived from the CMIP6 model projections.

The objective of this study is to partition changes in mean climate *state* versus *variability* to understand how crucial US Midwest agricultural productivity, as well as carbon and energy fluxes and their feedbacks to climate will be affected by 21st‐century climate change. We treat the combined agricultural productivity and carbon and energy fluxes and their feedbacks to climate as the “vulnerability” of agroecosystems. We used an Advanced WEather GENerator (AWE‐GEN) (Ivanov, Bras, and Curtis 2007) to produce future climate forcings (Figure 1) that were then used to drive the state‐of‐the‐science Energy Exascale Earth System Model (E3SM) (Golaz et al. 2022; Burrows et al. 2020) in a factorial simulation design that examined the relative and interactive effects of changes in future climate mean versus variability on US Midwest agroecosystems.

## 2 Material and Methods

### 2.1 Study Domain

The study area is the US Midwest region within North America covering 1.6 million km^2^ (Figure S1). This region was selected as it is intensively used for agricultural production and contains 36% of the United States cropland that produces 25% of national agricultural products (USDA 2017). The primary crops grown in this region are corn and soybean, both of which have been calibrated and validated in E3SM (Sinha et al. 2023).

### 2.2 Generating Climate Forcings

A crucial problem with partitioning the effects of changes in climate mean versus variability is that ESMs are known to be much stronger at predicting the former than the latter (Lee et al. 2021). To address this problem, we used an hourly weather generator, AWE‐GEN (Advanced WEather GENerator) (Ivanov, Bras, and Curtis 2007) to (1) downscale model outputs from Coupled Model Intercomparison Project Phase 6 (CMIP6) based on the methodology developed by Fatichi, Ivanov, and Caporali (2011) and (2) impose a given mean and/or variability change on grid‐cell‐scale climate. The weather generator is capable of reproducing characteristics of hydroclimatic variables of any given location at an hourly time scale while preserving the physical relationship among these variables (Ivanov, Bras, and Curtis 2007) and has been previously used in a stochastic downscaling framework to downscale climate forcing from global climate model projections to site level (Fatichi, Ivanov, and Caporali 2011). We utilized hourly ERA5 observational data (Hersbach et al. 2020) at a 0.25° spatial scale from 1980 to 2009 to train AWE‐GEN's parameters for simulating realistic values of atmospheric pressure, precipitation, shortwave radiation, air temperature, cloud cover, and wind components for the study domain (Figure S2). These parameters were estimated for all grid cells in the study domain and can be used to reproduce the historical climate variables.

For simulating future climate, we perturbed the historical parameters based on factors‐of‐change derived from the CMIP6 model projections (Fatichi, Ivanov, and Caporali 2011). We used CMIP6 outputs from five ESMs that were bias‐adjusted and archived in the Inter‐Sectoral Impact Model Intercomparison Project (ISIMIP) (Lange and Büchner 2021) database: GFDL‐ESM4, IPSL‐CM6A‐LR, MPI‐ESM1‐2‐HR, MRI‐ESM2‐0, and UKESM1‐0‐LL. The factors of change were estimated for each month by comparing CMIP6 ESM projected temperature and precipitation for the future period (2070–2099) to their estimates for the control period (1980–2009). The factors of change are then used for modifying AWE‐GEN's observationally tuned parameters for simulating future climate. We applied additive factor of change for the mean monthly temperature (Figure S3) and multiplicative factor of change for the mean monthly precipitation (Figure S4). Since the ESMs' projected climate variability has large uncertainty (Lee et al. 2021), we examined the observed variability in daily temperature and precipitation for each month in the study region and in the whole of North America (Figure S5). The average ratio of the 95th quantile to the 50 quantile across all months for the study domain is 1.12 and 1.47 for temperature and precipitation, respectively, and 1.47 and 2.02 across the entire North America. We used the average ratio across all months for North America as a multiplier in our study to represent an increase in climate variability in the future. As a result, we used a factor of 1.5 for increasing future temperature variability and used a factor of 2.0 for increasing future precipitation variability (Figure 1b and Figure S6b).

The AWE‐GEN estimates future climate individually for each grid cell and does not account for spatial coherence. As a result, our analysis examines the impacts of these forcings at the grid level. The inclusion of spatial coherence in climate forcing will amplify the reported impacts at a regional scale but not the direction of change for various fluxes/pools or the feedback of agroecosystems to the climate as reported in this study.

The end result was nine 30‐year time series of climate forcing data for each of 2829 $0.25^{{}^{\circ}}\times 0.25^{{}^{\circ}}$ grid cells in the 1.6 million km^2^ study region: Control set with current conditions; a set with change in temperature mean ($\Delta T_{m}$); set with change in temperature variability ($\Delta T_{v}$); set with change in temperature mean and variability ($\Delta T_{m+v}$); set with change in precipitation mean ($\Delta P_{m}$); set with change in precipitation variability ($\Delta P_{v}$); set with change in precipitation mean and variability ($\Delta P_{m+v}$); set with change in temperature and precipitation means ($\Delta T_{m}+\Delta P_{m}$); set with change in temperature and precipitation variability ($\Delta T_{v}+\Delta P_{v}$); and set with change in temperature and precipitation means and variability ($\Delta T_{m+v}+\Delta P_{m+v}$). Averaged across the spatial domain, the future forcing resulted in 7°C higher mean temperature in $\Delta T_{m}$, seasonal shift in rainfall with the annual amount unchanged in $\Delta P_{m}$, and 1.2°C higher temperature variability in $\Delta T_{v}$ averaged across all model years (Figure 1b and Figure S6b). The 7°C higher mean temperature in our forcing is in line with the temperature projection for the US Midwest in the Fifth National Climate Assessment Report. This report predicts that United States will warm more than the global average and for a 4°C global warming an increase of 5°C–6°C is predicted for the US Midwest (Marvel et al. 2023). AWE‐GEN estimated future forcings are less sensitive to changes in precipitation. This may be because AWE‐GEN estimation of historical precipitation deviates more from the observations than AWE‐GEN estimated temperature (Figure S2).

### 2.3 E3SM Land Model

The E3SM land model version 2 (ELMv2) (Golaz et al. 2022) includes active biogeochemistry components that simulate the observed dynamics of the terrestrial carbon cycle well (Burrows et al. 2020). The advantage of using ELM for this study, as opposed to a crop or ecosystem model, is that ELM allows for interactive feedback between various carbon cycle and biogeochemical processes. ELM utilizes multiple prognostic pools for carbon, nitrogen, and phosphorus and includes active phenology for both natural vegetation and crops. Plant productivity and soil microbial activity are limited by nutrient availability that couples the carbon, nitrogen, and phosphorus cycles (Burrows et al. 2020).

Importantly, we used a version of ELM optimized for agricultural modeling. As opposed to the simplistic representation of crops as natural grasses used by many previous ESMs (Bonan and Doney 2018), this version of ELM explicitly represents annual and perennial crops (Drewniak et al. 2013; Sinha et al. 2022). It also simulates various growth phases of crops, accounts for agricultural fertilizer application, allows for cropland fraction to change over time, and can simulate realistic crop rotation. Additionally, corn, soybean, *Miscanthus*, and switchgrass crops have been recently calibrated and validated for the US Midwest region (Sinha et al. 2022, 2023).

### 2.4 ELM Simulation and Analysis

We ran the ELM for 200 years in accelerated spin‐up mode, followed by 600 years in non‐accelerated spin‐up mode, and 130 years in transient mode from 1850 to 1979. The accelerated and non‐accelerated spin‐up simulations are performed to bring the carbon pool to 1850 equilibrium conditions (Thornton and Rosenbloom 2005) while the transient simulation accounts for land use change, CO_2_ increase, nitrogen deposition, and change in climate forcings between 1850 and 1979, resulting in realistic 1980 land and climate conditions for the start of the experimental simulations. We used Global Soil Wetness Project Phase 3 (GSWP3) meteorological forcing data for the spin‐up simulations and the transient simulations from 1850 to 1979. The GSWP3 forcing data is available from 1901 to 2014 and therefore for simulations periods before 1901, spin‐ups and transient simulations from 1850 to 1900, GSWP3 forcing data from 1901 to 1920 was recycled. The transient run from 1901 to 1979 used the GSWP3 data from this period. The transient simulation from 1980 to 2009 and the future periods used AWE‐GEN generated forcings for their respective periods (Table 1). The transient simulations used land use time series for capturing land use change and cropland expansion from 1850 onwards. The root distribution and depth are identical for all crops in this simulation.

**TABLE 1:** Simulation details—meteorological forcings, time period, surface dataset, and land use time series used.

Set	Description	Time period	Surface dataset	Land use time series
Ad spin‐up	GSWP3 forcing	0–200	1850	—
Final spin‐up	GSWP3 forcing	201–800	1850	—
Historical	GSWP3 forcing	1850–1979	1850	1850–1979
Control	AWE‐GEN generated forcing	1980–2009	1980	1980–2009
$\Delta T_{m}$	Temperature mean changed	2070–2099	1980	1980–2009
$\Delta T_{v}$	Temperature variability changed	2070–2099	1980	1980–2009
$\Delta T_{m+v}$	Temperature mean + variability changed	2070–2099	1980	1980–2009
$\Delta P_{m}$	Precipitation mean changed	2070–2099	1980	1980–2009
$\Delta P_{v}$	Precipitation variability changed	2070–2099	1980	1980–2009
$\Delta P_{m+v}$	Precipitation mean + variability changed	2070–2099	1980	1980–2009
$\Delta T_{m}+\Delta P_{m}$	Temp and precip means changed	2070–2099	1980	1980–2009
$\Delta T_{v}+\Delta P_{v}$	Temp and precip variability changed	2070–2099	1980	1980–2009
$\Delta T_{m+v}+\Delta P_{m+v}$	Temp and precip means + variability changed	2070–2099	1980	1980–2009

Since the objective of the study was to examine the impacts of change in mean climate and climate variability on agricultural fluxes, we used the land use time series for the historical control period (1980–2009) to represent land use change for the future years (2070–2099) as well (Table 1). Our land use time series ends in 2009 and avoids most of the surge in corn ethanol production that started in 2008 after the enactment of the Renewable Fuel Standard (Lark et al. 2022). Although the control and future simulations were performed for 30 years (1980–2009 and 2070–2099, respectively), only the last 20 years of the simulation results were used for quantifying the differences between control and future periods (1990–2009 for the control set and 2080–2099 for the future sets). This was done to avoid the sharp jump in carbon fluxes and pools that was caused by the sudden change in the forcing from the end of the historical period (2009) to the start of the future period (2070).

We quantified the change in various output variables across all grid cells in the domain by estimating normalized mean error (NME). For estimating NME, the mean variable value was estimated for each grid cell across the entire simulation period and the mean error (ME) was estimated by comparing future sets (2080–2099) to the control set (1990–2009). NME was estimated by first averaging the ME across the spatial domain and then normalizing it by the mean of the control set.

To assess the robustness of our findings, we first estimated the significance of change between control (1990–2009) and future scenarios (2080–2099) by performing a two‐sided *t*‐test at the 95% confidence level for each grid cell. In addition, we performed a sensitivity analysis experiment to examine if the observed impact can be attributed to climate's internal variability or the naturally occurring variance in the climate system. For this sensitivity analysis we generated four new forcings for increase in temperature mean ($\Delta T_{m}$) by using a different random number for generating the forcing. Since sensitivity analysis experiments are computationally expensive, we performed them for only one of our simulation set.

## 3 Results

### 3.1 Carbon Fluxes and Pools

**FIGURE 2:**
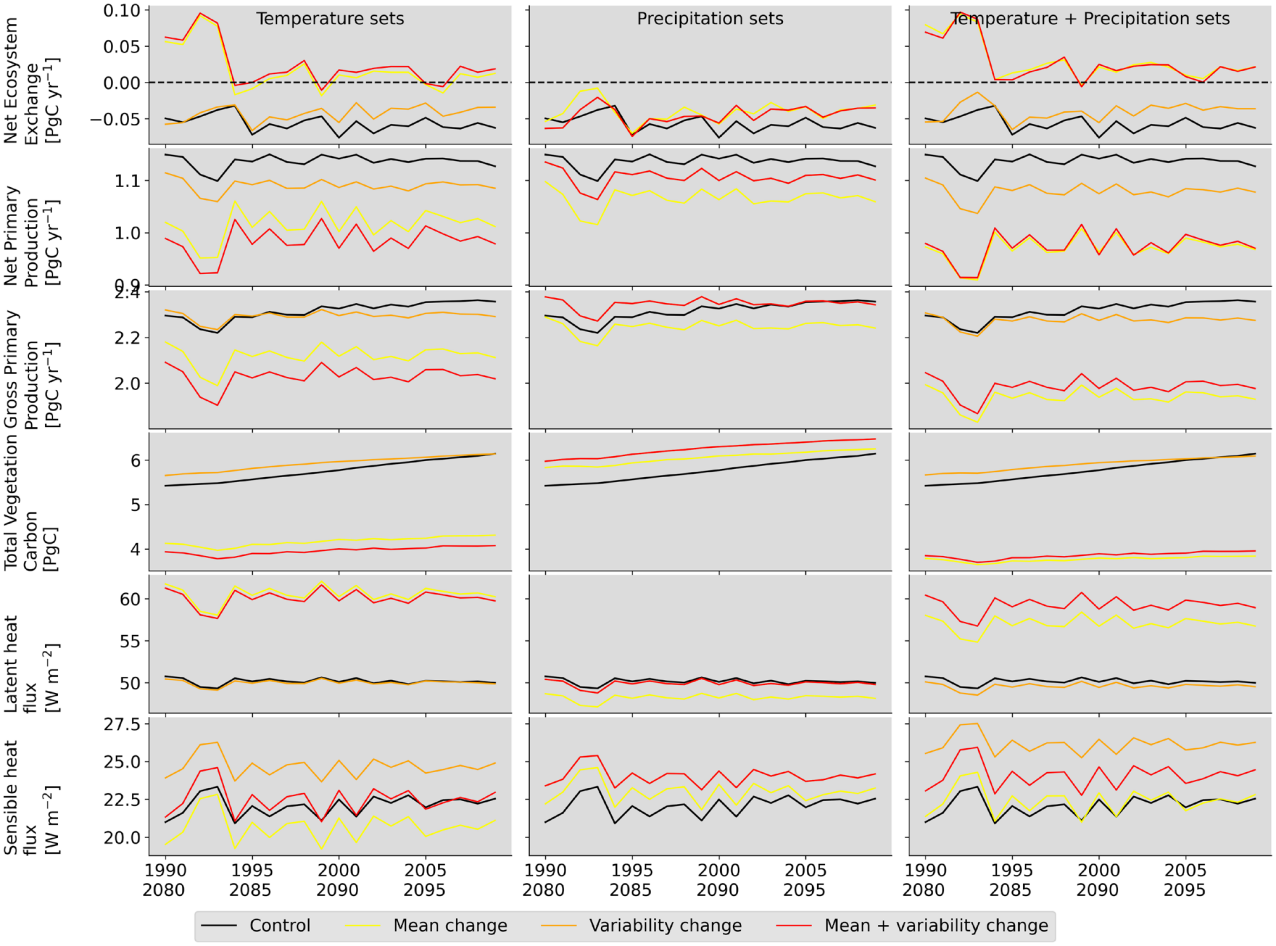
Time series of various carbon and energy fluxes the entire study region for: Control set from 1990 to 2009 (black color), and future sets from 2080 to 2099. Sets with modification to temperature mean and/or variability shown in left column ($\Delta T_{m}$, $\Delta T_{v}$, and $\Delta T_{m+v}$), sets with modification to precipitation mean and/or variability shown in middle column ($\Delta P_{m}$, $\Delta P_{v}$, and $\Delta P_{m+v}$), and sets with modification to both temperature and precipitation means and/or variability shown in right column ($\Delta T_{m}+\Delta P_{m}$, $\Delta T_{v}+\Delta P_{v}$, and $\Delta T_{m+v}+\Delta P_{m+v}$). Future sets with change in temperature and/or precipitation means shown in yellow color ($\Delta T_{m}$, $\Delta P_{m}$, and $\Delta T_{m}+\Delta P_{m}$). Future sets with change in temperature and/or precipitation variability shown in orange color ($\Delta T_{v}$, $\Delta P_{v}$, and $\Delta T_{v}+\Delta P_{v}$). Future sets with change in temperature and/or precipitation means and/or variability are shown in red color ($\Delta T_{m+v}$, $\Delta P_{m+v}$, and $\Delta T_{m+v}+\Delta P_{m+v}$). In the second column, the $\Delta P_{v}$ line, in orange, falls underneath the $\Delta P_{m+v}$ line in red and is therefore not visible.

We found that increases in temperature mean and variability interact nonlinearly in terms of their impacts of carbon fluxes and pools. The nonlinear interaction implies that the combined effect cannot be predicted by knowing only the individual effects. An increase in temperature ($\Delta T_{m}$) mean lowers plant productivity and results in loss of carbon at both regional and grid scales (Figure 2 and Figure S7). The compounded impact of temperature mean and variability changes ($\Delta T_{m+v}$) lowers carbon fluxes and storage pools more than simple summation of the individual impacts (Table S1). Averaging over the last 20 years of the simulations sets, regional gross primary productivity (GPP), that represents carbon taken‐up by plants, decreases by 9%, 1%, and 13% due to change in mean temperature ($\Delta T_{m}$), change in temperature variability ($\Delta T_{v}$), and changes in both temperature mean and variability ($\Delta T_{m+v}$), respectively. The vegetation carbon pool is reduced due to change in mean temperature (28%) but increases due to change in temperature variability (3%). Our findings are robust across an ensemble of simulations, that is, the projected changes are greater than that due to natural climate variability (Figure S8).

The combined impact of increase in both temperature mean and variability ($\Delta T_{m+v}$), however, results in vegetation carbon pool decreasing by 31%, reflecting the consequence of extreme high temperatures. Compared to the control set, the projected decrease in GPP and vegetation carbon pool are statistically significant for more than 78% of grid cells in the study domain for scenarios with change in temperature mean ($\Delta T_{m}$) and change in both temperature mean and temperature variability ($\Delta T_{m+v}$) (Figure S9 and Table S2). We also find that statistically significant decrease in GPP and vegetation carbon pool occurs in 36% and 72% of the grid cells, respectively, due to the combined impact of increase in both temperature mean and variability ($\Delta T_{m+v}$) compared to only increase in temperature mean ($\Delta T_{m}$) (Figure S10), representing the non‐linear interactions between mean and variability.

The impact of change in mean precipitation on carbon fluxes and pools is lower than that of temperature (Figure 2), because precipitation shifts seasonally with minimal change in annual total (Figure S6a). At the regional scale, GPP experiences slight reduction with change in precipitation mean ($\Delta P_{m}$) (3%) and an increase with change in precipitation variability ($\Delta P_{v}$) (1.3%) and change in both precipitation mean and variability ($\Delta P_{m+v}$) (1.3%) (Table S1). The vegetation carbon pool increases for future change in precipitation mean and/or variability compared to the control run (5%, 9%, and 9%). The projected increase in vegetation carbon pool was statistically significant in more than 70% of the grid cells for simulations with change in precipitation mean and/or variability ($\Delta P_{m}$, $\Delta P_{v}$, and $\Delta P_{m+v}$); GPP changes were less widespread (Table S2). However, compared to $\Delta P_{m}$, statistically significant change occurs in GPP and vegetation carbon pool in approximately 50% of the grid cells in $\Delta P_{m+v}$ (Figure S11).

Joint change in temperature and precipitation means or variability lowers the carbon fluxes and storage pools more than summation of individual components. At the regional scale, GPP decreases by 16% when both temperature and precipitation means were changed ($\Delta T_{m}+\Delta P_{m}$) while it reduces by 9% when only temperature mean ($\Delta T_{m}$) and 3% when only precipitation mean ($\Delta P_{m}$) were changed (Table S1). The vegetation carbon pool decreases by 35% for $\Delta T_{m}+\Delta P_{m}$ while it decreases by 28% for $\Delta T_{m}$ and increased by 5% for $\Delta P_{m}$. Similarly, GPP decreases by 1.7% when both temperature and precipitation variability are changed ($\Delta T_{v}+\Delta P_{v}$) but is reduced by 0.9% when only temperature variability was changed ($\Delta T_{v}$) and increases by 1.3% with precipitation variability change ($\Delta P_{v}$). The vegetation carbon pool increases by 2.4%, 2.8%, 8.5% for $\Delta T_{v}+\Delta P_{v}$, $\Delta T_{v}$ and $\Delta P_{v}$, respectively.

Overall, the scenario with change in both temperature and precipitation means leads to the largest reduction in carbon fluxes and carbon pools, more than the scenario that considers all four factors, that is, change in temperature and precipitation means and variability ($\Delta T_{m+v}+\Delta P_{m+v}$). This is because change in precipitation variability results in an increase in carbon pool and storage ($\Delta P_{v}$). Together the change in temperature and precipitation means ($\Delta T_{m}+\Delta P_{m}$) reduces GPP by 16% and vegetation carbon pool by 35% and this reduction is statistically significant for more than 85% of grid cells (Tables S1 and S2).

**FIGURE 3:**
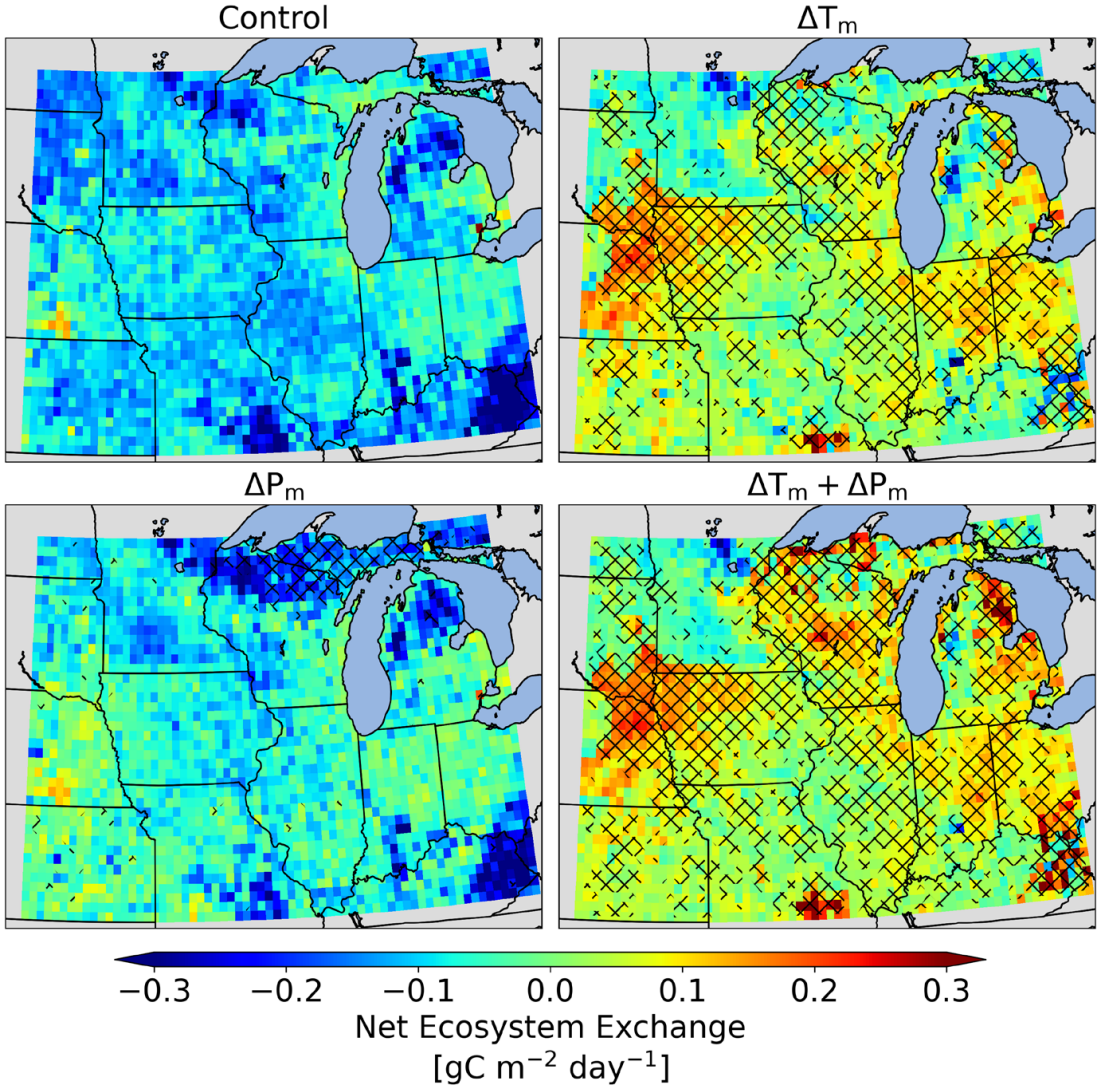
Total annual carbon balance (net ecosystem exchange) for the control, $\Delta T_{m}$, $\Delta P_{m}$, and $\Delta T_{m}+\Delta P_{m}$ simulations over the last 20 years of simulation. Stippling indicate significant change between control (1990–2009) and future scenarios (2080–2099). Map lines delineate study area and do not necessarily depict accepted regional boundaries.

At a local (grid cell) scale, the increased temperature also causes carbon fluxes to decrease over most of the US Midwest, in both agriculturally intensive regions and non‐agriculture dominated regions. Comparing normalized mean error (NME) (see Methods) across various output variables reveals that the largest reduction due to an increase in temperature occurs in vegetation carbon (Figure 3), while nitrogen and phosphorus mineralization increase at higher temperatures. Similar to the findings at the regional scale, changes in both temperature and precipitation means lead to the largest reduction in carbon fluxes and storage pools.

### 3.2 Feedback to the Climate System

**FIGURE 4:**
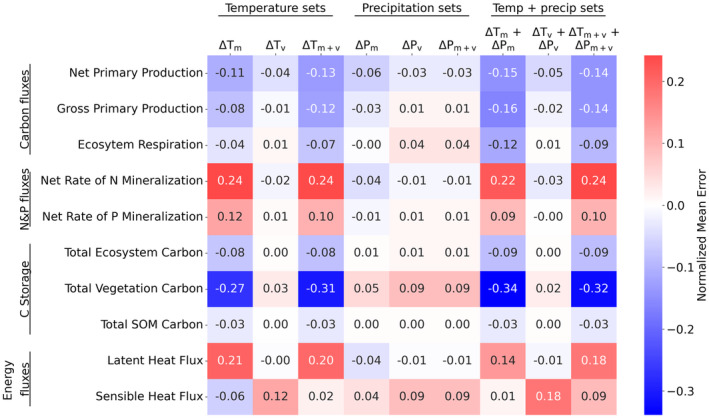
Normalized mean error (NME) for various climatic and meteorological forcing variables. For estimating NME, the mean variable value was estimated for each grid cell across the entire simulation period and the mean error (ME) was estimated by comparing future sets to the control set of simulations. NME was estimated by first averaging the ME across the spatial domain and then normalizing it by the mean of the control set.

Mean temperature changes cause agriculturally intensive regions to become a carbon source at regional (Figure 2 top‐row) and local (grid cell) scale (Figure 4). Hot conditions shift and shorten the crop growing cycle resulting in plant dying off much sooner (Figure S12e,f). This can cause the land‐atmosphere carbon balance (net ecosystem exchange, NEE) from croplands to become positive and thus a source to the atmosphere (Figure S2d). This transition from carbon sink to source was consistent for all scenarios with increased temperature means and was similar in magnitude regardless of changes in precipitation mean or variability, or temperature variability ($\Delta T_{m}$, $\Delta T_{m+v}$, $\Delta T_{m}+\Delta P_{m}$, and $\Delta T_{m+v}+\Delta P_{m+v}$), implying that this change can be attributed primarily to increase in temperature mean (Figure 2). Averaging over the last 20 years of the simulations sets, NEE decreases by 133%, 133%, 150%, and 150% due to change in mean temperature ($\Delta T_{m}$), changes in both temperature mean and variability ($\Delta T_{m+v}$), change in temperate and precipitation means ($\Delta T_{m}+\Delta P_{m}$), and change in temperature and precipitation means and variability ($\Delta T_{m+v}+\Delta P_{m+v}$), respectively. The change in mean precipitation ($\Delta P_{m}$) lowers NEE by 33% in the entire US Midwest region; however, the impact on the agriculturally intensive regions in the corn belt are larger and are counterbalanced by an increase in NEE in the forested regions of northern Wisconsin, Michigan, and Kentucky (Figure 4). The forested regions are projected to experience a similar opposite impact on NEE for change in mean temperature ($\Delta T_{m}$) as well as for GPP for both $\Delta T_{m}$ and $\Delta P_{m}$ sets (Figure S9).

### 3.3 Energy Fluxes Between the Land and Atmosphere

Energy fluxes, balance, and partitioning were all strongly affected as well. Temperature mean changes increased latent heat flux, while temperature variability change contributed to significant increase in sensible heat flux. An increase in temperature mean results in latent heat flux increasing by more than 20% in $\Delta T_{m}$ and $\Delta T_{m+v}$ and this increase is statistically significant for 99% of the grid cells ($\Delta T_{m}$ and $\Delta T_{m+v}$ in Figures 2 and 3 and Tables S2 and S3). An increase in precipitation mean ($\Delta P_{m}$) lowers the latent heat flux by 4% and this reduction is statistically significant for more than 70% of the grid cells. Conversely, scenarios with change in temperature variability and precipitation variability ($\Delta T_{v}$ and $\Delta P_{v}$) are projected to have minimal impact on latent heat flux. Sensible heat increases from 9% to 18% for sets with change in temperature variability ($\Delta T_{v}$, $\Delta T_{v}+\Delta P_{v}$, and $\Delta T_{m+v}+\Delta P_{m+v}$) and this change is statistically significant in more than 72% of the grid cells in these sets (Figure 3 and Tables S2 and S3). For sets other than $\Delta T_{v}$, $\Delta T_{v}+\Delta P_{v}$, and $\Delta T_{m+v}+\Delta P_{m+v}$, the change in sensible heat is statistically significant for a smaller fraction of grid cells in the domain (25%–64% of grid cells).

### 3.4 Crop Yield and Productivity

**FIGURE 5:**
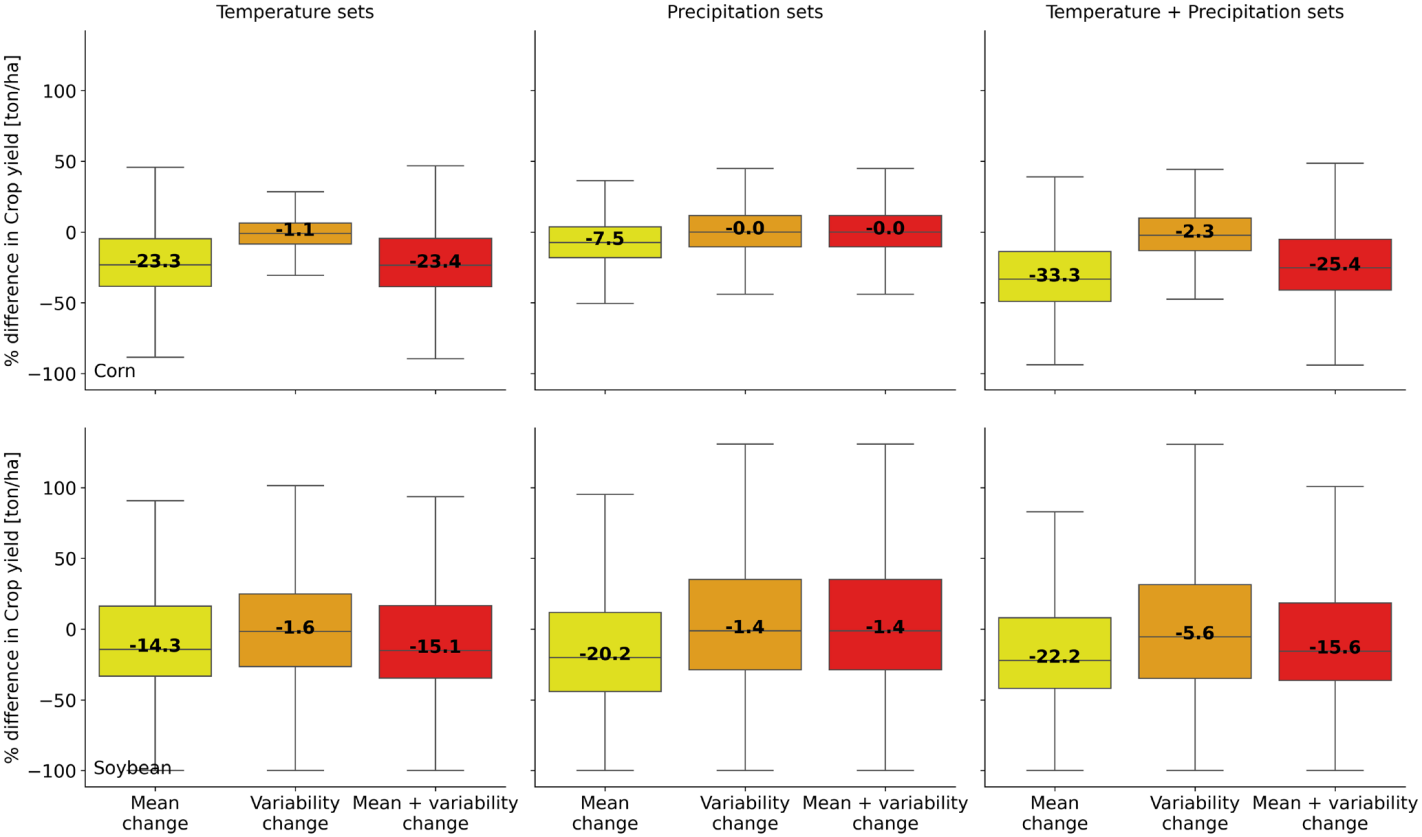
Distribution of percentage difference in yield between future sets and control set for corn and soybean. The boxplots show the distribution of percentage difference in yield across all grid cells and over the last 20‐year period. The number in the center of the boxplots represents the median value of the boxplot. The outlier points are removed to increase the plot's readability.

Consistent with decades of crop observations (Zhao et al. 2017), higher temperature lowers corn yield more than soybean yield while change in precipitation lowers soybean yield more than corn yield (Figure 5 and Figure S13). Higher temperatures, despite an earlier start of the growing season, reduce the growing period of crops, and in particular, the duration spent in the grain fill phase (Figures S12 and S14); this is consistent with ref. Ruiz‐Vera et al. (2018) that elevated temperatures accelerate both the vegetative and reproductive growth phases of corn and soybean. This results in −33% and −22% median reduction in yield for corn and soybean, respectively, compared to the control period. The reduction in yield, caused by higher temperature is larger for corn than for soybean and also occurs in a larger fraction of grid cells with corn than in the grid cells with soybean (Figure 5). The greater reduction in corn yield is caused by a larger reduction in its grain fill phase duration due to lower growing degree day requirements compared to soybean (Figure S14). The change in precipitation amount has a negligible impact on grain fill phase duration; however, as the start of various growth cycles depends almost exclusively on temperature. We infer that change in precipitation impacts crop yield via its impact on plant water stress and plant nitrogen uptake.

Dry conditions result in a loss of productivity for both crops (Figure S15). The impact of change in precipitation is less when examining annual averages instead of at smaller timescales due to the projected change in precipitation resulting in a seasonal shift that has a minimal impact on the annual mean (Figure S6). Similarly, the precipitation impacts are reduced when estimating spatial means, as AWE‐GEN generates independent forcing for each grid cell that does not account for spatial correlation. Therefore, we examined the impact of dry conditions at a monthly to seasonal scale and at a grid scale level. Reduced rainfall causes plant productivity to decrease as compared to the control run (Figure S15b,e). Dry conditions also increase plant water stress, reduce plant nitrogen uptake, and cause nitrogen limitation during the grain fill phase that results in a reduction in crop yield.

## 4 Discussion

### 4.1 Agroecosystems' Vulnerability to Climate Change

Ongoing and future climate change is altering temperature and precipitation patterns in many global agroecosystems (Lobell, Schlenker, and Costa‐Roberts 2011; Marvel et al. 2023; Bolster et al. 2023), including in the US Midwest region that is globally important in agricultural commodities (USDA 2017) and the focus of this study. Previous studies have found that precipitation amount (Kumar et al. 2004; Guan et al. 2015) and increasing temperature trends (Lobell, Schlenker, and Costa‐Roberts 2011; Asseng et al. 2015) can both greatly impact crop yield. Elevated temperatures lower soil carbon content (Black et al. 2017) and increase carbon fluxes from croplands (Gao et al. 2022); increased variability and the attendant risk of extreme events such as drought may reduce carbon storage in natural ecosystems (Rastetter et al. 2023).

Our agroecosystem‐focused study is consistent with these previous results but, importantly, finds non‐linear interactive effects of mean and variability changes. Such effects are likely caused by extremely high temperatures (Schlenker and Roberts 2009) or a combination of hot and dry conditions (Hatfield and Prueger 2015). After the temperature threshold for plant growth is reached, plant productivity declines sharply and non‐linearly (Schlenker and Roberts 2009), meaning that the reduction in plant growth will be larger at higher temperatures than the combined reduction at lower temperatures. Hot and dry conditions together are shown to further lower plant productivity (Hatfield and Prueger 2015). Similar nonlinear responses to temperature increases have been reported for soil respiration (Qi, Xu, and Wu 2002). Our analysis does not account for the spatial coherence of droughts (see Methods), and thus provides a lower bound—i.e., future impacts at regional scales might be considerably worse.

Agroecosystems will have a positive feedback to the climate system with increased warming, as agricultural land converts from being a carbon sink to a carbon source due to increased temperature (Figure 4 and Table S1). The resulting increased atmospheric carbon dioxide concentrations will further amplify warming, consistent with many terrestrial and soil‐focused modeling studies (Canadell et al. 2023) and observations (Melillo et al. 2002, 2011, 2017). Agroecosystems' climate feedback can, however, be negative at local scales, since our analysis reveals that warming will decrease the Bowen ratio (the ratio of sensible to latent heat flux) that may cause local cooling. Coupled land‐atmosphere model simulations will be necessary to fully understand how local, regional, and global climate will be impacted by these opposing and complex feedbacks from agroecosystems. How land use change will interact with climate in agroecosystems is another important area of research (Findell et al. 2017; Lawrence et al. 2016), as it is a factor that we did not include in this analysis.

A crucial test for the ELM used here is its grid‐scale performance relative to the known behavior of agricultural systems under changing environmental conditions. Dry conditions impact the yield of both corn and soybean due to water stress but via different mechanisms. Corn productivity is directly affected by water stress, and this is captured in ELM by regulating photosynthesis through the water stress or transpiration beta parameter (BTRAN). BTRAN determines soil moisture limitation on plant transpiration and photosynthesis using a curvilinear fit to soil water potential controls on stomata that are specific to each crop (Oleson et al. 2013). The drought resistance characteristics of soybean are captured in ELM by increasing its BTRAN factor. However, water stress limits soybean's ability to fix atmospheric nitrogen, and drier soil also lowers nitrogen mineralization rates. Together, these two processes greatly limit nitrogen availability for soybean, resulting in yield reduction. In contrast, the impact of reduced soil nitrogen mineralization is felt less by corn due to higher fertilizer applied to it compared with soybean. Higher temperatures, however, increase nitrogen mineralization and can alleviate or reduce nitrogen limitation for crop growth.

Our findings have implications for policies aimed at mitigating the climate feedback, local‐scale crop production, soil carbon sequestration, conservation of existing soil carbon stocks, and soil health risks of climate change. Developing such policies based only on mean climate change while omitting the impact of change in climate variability will likely underestimate the full extent of the impact and thereby reduce the effectiveness of the mitigation policies. In particular, agricultural practices of irrigation, fertilizer application, and crop residue management can be used to counteract the loss of productivity due to climate change. For example, Li et al. (2020) found that cooling benefits alone from irrigation have a significant impact on increasing yield. Similarly, Levis et al. (2018) and Obata and Tsujino (2024) found increased irrigation and ${\mathrm{CO}}_{2}$ fertilization under future scenarios can offset yield losses from temperature increase, and in some cases result in yield gains. However, increased water demand under these irrigation scenarios will be much larger when non‐linear interactive effects of mean and variability changes are considered and will likely compete with water demand for other sectors. Therefore, such a study should couple ESMs with socioeconomic models that can simulate interactions and interdependencies among human and natural systems.

For these reasons, we conclude that coupled human‐earth system model analyses (Collins et al. 2015; Thornton et al. 2017), in which economic and social policies drive changes in emissions and land use in response to climate, will be crucial for robustly assessing the mitigation potential of agricultural management. Our work shows that it is crucial that such policies account for the combined impact of change in both mean climate state and its variability as globally important agroecosystems confront 21st‐century climate change.

## Author Contributions

This study was designed by E.S. and B.B.‐L. D.X. provided guidance and feedback on the use of AWE‐GEN, and K.A.M. and B.A.D. contributed insights and ideas based on their expertise in biogeochemistry and crop modeling. E.S. performed all data processing, model setup, computational runs, and results analysis. E.S. led the writing of the manuscript with significant contributions from all authors.

## Conflicts of Interest

The authors declare no conflicts of interest.

## Supporting information


Data S1.

Data S2.

## Data Availability

The data and code that support the findings of this study are openly available in Zenodo at http://doi.org/10.5281/zenodo.14675093 and http://doi.org/10.5281/zenodo.14682898, respectively. ERA5 observational data were obtained from the Copernicus Climate Change Service (C3S) Climate Data Store (CDS) at http://doi.org/10.24381/cds.adbb2d47. CMIP6 outputs were obtained from the Inter‐Sectoral Impact Model Intercomparison Project (ISIMIP) database via Zenodo at https://doi.org/10.5281/zenodo.4686991. The E3SM model (ELMv2) can be accessed from DOE CODE at https://doi.org/10.11578/E3SM/dc.20210927.1 and Github at https://github.com/E3SM‐Project/E3SM/releases/tag/v2.0.0.

## References

[gcb70064-bib-0001] An, Z. , E. W. Bork , D. Olefeldt , C. N. Carlyle , and S. X. Chang . 2022. “Simulated Heat Wave Events Increase CO_2_ and N_2_O Emissions From Cropland and Forest Soils in an Incubation Experiment.” Biology and Fertility of Soils 58, no. 7: 789–802. 10.1007/s00374-022-01661-w.

[gcb70064-bib-0002] Anderegg, W. R. , A. T. Trugman , G. Badgley , et al. 2020. “Climate‐Driven Risks to the Climate Mitigation Potential of Forests.” Science 368, no. 6497: eaaz7005. 10.1126/science.aaz7005. 32554569

[gcb70064-bib-0003] Asseng, S. , F. Ewert , P. Martre , et al. 2015. “Rising Temperatures Reduce Global Wheat Production.” Nature Climate Change 5, no. 2: 143–147. 10.1038/nclimate2470.

[gcb70064-bib-0004] Black, C. K. , S. C. Davis , T. W. Hudiburg , C. J. Bernacchi , and E. H. DeLucia . 2017. “Elevated CO_2_ and Temperature Increase Soil C Losses From a Soybean–Maize Ecosystem.” Global Change Biology 23, no. 1: 435–445. 10.1111/gcb.13378. 27252041

[gcb70064-bib-0005] Bolster, C. , R. Mitchell , A. Kitts , et al. 2023. “Agriculture, Food Systems, and Rural Communities.” Fifth National Climate Assessment. 10.7930/NCA5.2023.CH11.

[gcb70064-bib-0006] Bonan, G. B. , and S. C. Doney . 2018. “Climate, Ecosystems, and Planetary Futures: The Challenge to Predict Life in Earth System Models.” Science 359, no. 6375: eaam8328. 10.1126/science.aam8328. 29420265

[gcb70064-bib-0007] Burrows, S. , M. Maltrud , X. Yang , et al. 2020. “The Doe e3sm v1. 1 Biogeochemistry Configuration: Description and Simulated Ecosystem‐Climate Responses to Historical Changes in Forcing.” Journal of Advances in Modeling Earth Systems 12, no. 9: e2019. 10.1029/2019MS001766.

[gcb70064-bib-0008] Canadell, J. G. , P. M. Monteiro , M. H. Costa , et al. 2023. “Intergovernmental Panel on Climate Change (IPCC). Global Carbon and Other Biogeochemical Cycles and Feedbacks.” In Climate Change 2021: The Physical Science Basis. Contribution of Working Group I to the Sixth Assessment Report of the Intergovernmental Panel on Climate Change, edited by V. Brovkin , 673–816. Cambridge.

[gcb70064-bib-0009] Collins, W. D. , A. P. Craig , J. E. Truesdale , et al. 2015. “The Integrated Earth System Model Version 1: Formulation and Functionality.” Geoscientific Model Development 8, no. 7: 2203–2219. 10.5194/gmd-8-2203-2015.

[gcb70064-bib-0010] Crippa, M. , E. Solazzo , D. Guizzardi , F. Monforti‐Ferrario , F. N. Tubiello , and A. Leip . 2021. “Food Systems Are Responsible for a Third of Global Anthropogenic Ghg Emissions.” Nature Food 2, no. 3: 198–209. 10.1038/s43016-021-00225-9. 37117443

[gcb70064-bib-0011] Drewniak, B. , J. Song , J. Prell , V. Kotamarthi , and R. Jacob . 2013. “Modeling Agriculture in the Community Land Model.” Geoscientific Model Development 6, no. 2: 495–515. 10.5194/gmd-6-495-2013.

[gcb70064-bib-0012] Eyring, V. , S. Bony , G. A. Meehl , et al. 2016. “Overview of the Coupled Model Intercomparison Project Phase 6 (CMIP6) Experimental Design and Organization.” Geoscientific Model Development 9, no. 5: 1937–1958. 10.5194/gmd-9-1937-2016.

[gcb70064-bib-0013] FAO . 2024. FAOSTAT Food and Agriculture Organization of the United Nations. FAO.

[gcb70064-bib-0014] Fatichi, S. , V. Y. Ivanov , and E. Caporali . 2011. “Simulation of Future Climate Scenarios With a Weather Generator.” Advances in Water Resources 34, no. 4: 448–467. 10.1016/j.advwatres.2010.12.013.

[gcb70064-bib-0015] Findell, K. L. , A. Berg , P. Gentine , et al. 2017. “The Impact of Anthropogenic Land Use and Land Cover Change on Regional Climate Extremes.” Nature Communications 8, no. 1: 989. 10.1038/s41467-017-01038-w. PMC5651924 29057878

[gcb70064-bib-0016] Gao, H. , H. Tian , Z. Zhang , and X. Xia . 2022. “Warming‐Induced Greenhouse Gas Fluxes From Global Croplands Modified by Agricultural Practices: A Meta‐Analysis.” Science of the Total Environment 820: 153288. 10.1111/gcb.16627. 35066045

[gcb70064-bib-0017] Golaz, J.‐C. , L. P. Van Roekel , X. Zheng , et al. 2022. “The Doe E3SM Model Version 2: Overview of the Physical Model and Initial Model Evaluation.” Journal of Advances in Modeling Earth Systems 14, no. 12: 156. 10.1029/2022MS003156.

[gcb70064-bib-0018] Guan, K. , B. Sultan , M. Biasutti , C. Baron , and D. B. Lobell . 2015. “What Aspects of Future Rainfall Changes Matter for Crop Yields in West Africa?” Geophysical Research Letters 42, no. 19: 8001–8010. 10.1002/2015GL063877.

[gcb70064-bib-0019] Hatfield, J. L. , and J. H. Prueger . 2015. “Temperature Extremes: Effect on Plant Growth and Development.” Weather and Climate Extremes 10: 4–10. 10.1016/j.wace.2015.08.001.

[gcb70064-bib-0020] Hersbach, H. , B. Bell , P. Berrisford , et al. 2020. “The ERA5 Global Reanalysis.” Quarterly Journal of the Royal Meteorological Society 146, no. 730: 1999–2049. 10.1002/qj.3803.

[gcb70064-bib-0021] Ivanov, V. Y. , R. L. Bras , and D. C. Curtis . 2007. “A Weather Generator for Hydrological, Ecological, and Agricultural Applications.” Water Resources Research 43, no. 10: 5364. 10.1029/2006WR005364.

[gcb70064-bib-0022] Kumar, K. K. , K. R. Kumar , R. Ashrit , N. Deshpande , and J. W. Hansen . 2004. “Climate Impacts on Indian Agriculture.” International Journal of Climatology: A Journal of the Royal Meteorological Society 24, no. 11: 1375–1393. 10.1002/joc.1081.

[gcb70064-bib-0023] Lange, S. , and M. Büchner . 2021. “ISIMIP3b Bias‐Adjusted Atmospheric Climate Input Data.” https://www.isimip.org/gettingstarted/input‐data‐bias‐adjustment/.

[gcb70064-bib-0024] Lark, T. J. , N. P. Hendricks , A. Smith , et al. 2022. “Environmental Outcomes of the US Renewable Fuel Standard.” Proceedings of the National Academy of Sciences 119, no. 9: e2101084119. 10.1073/pnas.2101084119 PMC8892349 35165202

[gcb70064-bib-0025] Lawrence, D. M. , G. C. Hurtt , A. Arneth , et al. 2016. “The Land Use Model Intercomparison Project (Lumip) Contribution to CMIP6: Rationale and Experimental Design.” Geoscientific Model Development 9, no. 9: 2973–2998. 10.5194/gmd-9-2973-2016.

[gcb70064-bib-0026] Lee, J.‐Y. , J. Marotzke , G. Bala , et al. 2021. Future Global Climate: Scenario‐Based Projections and Near‐Term Information. Cambridge University Press.

[gcb70064-bib-0027] Lesk, C. , P. Rowhani , and N. Ramankutty . 2016. “Influence of Extreme Weather Disasters on Global Crop Production.” Nature 529, no. 7584: 84–87. 10.1038/nature16467. 26738594

[gcb70064-bib-0028] Levis, S. , A. Badger , B. Drewniak , C. Nevison , and X. Ren . 2018. “Clmcrop Yields and Water Requirements: Avoided Impacts by Choosing RCP 4.5 Over 8.5.” Climatic Change 146: 501–515. 10.1007/s10584-016-1654-9.

[gcb70064-bib-0029] Li, Y. , K. Guan , B. Peng , T. E. Franz , B. Wardlow , and M. Pan . 2020. “Quantifying Irrigation Cooling Benefits to Maize Yield in the US Midwest.” Global Change Biology 26, no. 5: 3065–3078. 10.1111/gcb.15002. 32167221

[gcb70064-bib-0030] Lobell, D. B. , W. Schlenker , and J. Costa‐Roberts . 2011. “Climate Trends and Global Crop Production Since 1980.” Science 333, no. 6042: 616–620. 10.1126/science.1204531. 21551030

[gcb70064-bib-0031] Marvel, K. , W. Su , R. Delgado , et al. 2023. “Climate Trends.” Fifth National Climate Assessment. 10.7930/NCA5.2023.CH2.

[gcb70064-bib-0032] Melillo, J. M. , S. Butler , J. Johnson , et al. 2011. “Soil Warming, Carbon–Nitrogen Interactions, and Forest Carbon Budgets.” Proceedings of the National Academy of Sciences 108, no. 23: 9508–9512. 10.1073/pnas.1018189108. PMC3111267 21606374

[gcb70064-bib-0033] Melillo, J. M. , S. D. Frey , K. M. DeAngelis , et al. 2017. “Long‐Term Pattern and Magnitude of Soil Carbon Feedback to the Climate System in a Warming World.” Science 358, no. 6359: 101–105. 10.1126/science.aan2874. 28983050

[gcb70064-bib-0034] Melillo, J. M. , P. Steudler , J. D. Aber , et al. 2002. “Soil Warming and Carbon‐Cycle Feedbacks to the Climate System.” Science 298, no. 5601: 2173–2176. 10.1126/science.1074153. 12481133

[gcb70064-bib-0035] Neumann, M. , V. Mues , A. Moreno , H. Hasenauer , and R. Seidl . 2017. “Climate Variability Drives Recent Tree Mortality in Europe.” Global Change Biology 23, no. 11: 4788–4797. 10.1111/gcb.13724. 28417562 PMC5633074

[gcb70064-bib-0036] Obata, A. , and H. Tsujino . 2024. “Earth System Model's Capability of Predicting Drought‐Induced Crop Failure.” Environmental Earth Sciences 83, no. 13: 417. 10.1007/s12665-024-11723-x.

[gcb70064-bib-0037] Oleson, K. W. , D. M. Lawrence , G. B. Bonan , et al. 2013. “Technical Description of Version 4.5 of the Community Land Model (CLM).” NCAR Technical Note, 257. 10.5065/D6RR1W7M.

[gcb70064-bib-0038] Poore, J. , and T. Nemecek . 2018. “Reducing Food's Environmental Impacts Through Producers and Consumers.” Science 360, no. 6392: 987–992. 10.1126/science.aaq0216. 29853680

[gcb70064-bib-0039] Qi, Y. , M. Xu , and J. Wu . 2002. “Temperature Sensitivity of Soil Respiration and Its Effects on Ecosystem Carbon Budget: Nonlinearity Begets Surprises.” Ecological Modelling 153, no. 142: 131. 10.1016/S0304-3800(01)00506-3.

[gcb70064-bib-0040] Rastetter, E. B. , K. L. Griffin , B. L. Kwiatkowski , and G. W. Kling . 2023. “Ecosystem Feedbacks Constrain the Effect of Day‐to‐Day Weather Variability on Land–Atmosphere Carbon Exchange.” Global Change Biology 29, no. 21: 6093–6105. 10.1111/gcb.16926. 37647012

[gcb70064-bib-0041] Rippey, B. R. 2015. “The US Drought of 2012.” Weather and Climate Extremes 10: 57–64. 10.1016/j.wace.2015.10.004.

[gcb70064-bib-0042] Ruiz‐Vera, U. M. , M. H. Siebers , D. Jaiswal , D. R. Ort , and C. J. Bernacchi . 2018. “Canopy Warming Accelerates Development in Soybean and Maize, Offsetting the Delay in Soybean Reproductive Development by Elevated CO_2_ Concentrations.” Plant, Cell & Environment 41, no. 12: 2806–2820. 10.1111/pce.13410. 30055106

[gcb70064-bib-0043] Schlenker, W. , and M. J. Roberts . 2009. “Nonlinear Temperature Effects Indicate Severe Damages to US Crop Yields Under Climate Change.” Proceedings of the National Academy of Sciences of the United States of America 106, no. 37: 15594–15598. 19717432 10.1073/pnas.0906865106 PMC2747166

[gcb70064-bib-0044] Sinha, E. , B. Bond‐Lamberty , K. V. Calvin , et al. 2023. “The Impact of Crop Rotation and Spatially Varying Crop Parameters in the E3SM Land Model (ELMv2).” Journal of Geophysical Research: Biogeosciences 128, no. 3: e2022JG. 10.1029/2022JG007187.

[gcb70064-bib-0045] Sinha, E. , K. V. Calvin , B. Bond‐Lamberty , et al. 2022. “Modeling Perennial Bioenergy Crops in the E3SM Land Model (ELMv2).” Journal of Advances in Modeling Earth Systems 15, no. 1: e2022M. 10.1029/2022MS003171.

[gcb70064-bib-0046] Thomey, M. L. , S. L. Collins , R. Vargas , et al. 2011. “Effect of Precipitation Variability on Net Primary Production and Soil Respiration in a Chihuahuan Desert Grassland.” Global Change Biology 17, no. 4: 1505–1515. 10.1111/j.1365-2486.2010.02363.x.

[gcb70064-bib-0047] Thornton, P. E. , K. Calvin , A. D. Jones , et al. 2017. “Biospheric Feedback Effects in a Synchronously Coupled Model of Human and Earth Systems.” Nature Climate Change 7, no. 7: 496–500. 10.1038/nclimate3310.

[gcb70064-bib-0048] Thornton, P. E. , and N. A. Rosenbloom . 2005. “Ecosystem Model Spin‐Up: Estimating Steady State Conditions in a Coupled Terrestrial Carbon and Nitrogen Cycle Model.” Ecological Modelling 189, no. 1–2: 25–48. 10.1016/j.ecolmodel.2005.04.008.

[gcb70064-bib-0049] Tubiello, F. N. 2019. “Greenhouse Gas Emissions due to Agriculture.” Encyclopedia of Food Security and Sustainability 3: 196–205. 10.1016/B978-0-08-100596-5.21996-3.

[gcb70064-bib-0050] Tubiello, F. N. , C. Rosenzweig , G. Conchedda , et al. 2021. “Greenhouse Gas Emissions From Food Systems: Building the Evidence Base.” Environmental Research Letters 16, no. 6: 65007. 10.1088/1748-9326/ac018e.

[gcb70064-bib-0051] USDA . 2017. “USDA National Agricultural Statistics Service, 2017 Census of Agriculture.” Technical Report, USDA, Washington, D.C., USA. www.nass.usda.gov/AgCensus.

[gcb70064-bib-0052] Zhao, C. , B. Liu , S. Piao , et al. 2017. “Temperature Increase Reduces Global Yields of Major Crops in Four Independent Estimates.” Proceedings of the National Academy of Sciences 114, no. 35: 9326–9331. 10.1073/pnas.1701762114. PMC5584412 28811375

[gcb70064-bib-0053] Zomer, R. J. , D. A. Bossio , R. Sommer , and L. V. Verchot . 2017. “Global Sequestration Potential of Increased Organic Carbon in Cropland Soils.” Scientific Reports 7, no. 1: 15554. 10.1038/s41598-017-15794-8. 29138460 PMC5686149

