# Supplementary material for: Interactions Between Climate Mean and Variability Drive Future Agroecosystem Vulnerability
Source: Glob Chang Biol. 2025 Feb 7;31(2):e70064. doi: 10.1111/gcb.70064 (PMC11803497; doi:10.1111/gcb.70064)
Supplement: Supplementary file 1 — Data S1. [file GCB-31-e70064-s002.pdf]

Supporting Information for "Interactions between climate mean and variability drive future agroecosystem vulnerability"

Eva Sinha[1], Donghui Xu[1], Kendalynn A. Morris[2], Beth A. Drewniak[3] Ben Bond-Lamberty[2]

1. Atmospheric, Climate, & Earth Sciences Division, Pacific Northwest National Laboratory, Richland, WA, United States;

2. Joint Global Change Research Institute, Pacific Northwest National Laboratory, College Park, MD, United States;

3. Environmental Science Division, Argonne National Laboratory, Lemont, IL, United States.

∗ Corresponding author; e-mail: eva.sinha@pnnl.gov.

Supplementary Figures and Tables

Contents of this file

1. Figures S1 to S16

References

[1] Hans Hersbach, Bill Bell, Paul Berrisford, Shoji Hirahara, András Horányi, Joaquín Muñoz-Sabater, Julien Nicolas, Carole Peubey, Raluca Radu, Dinand Schepers, et al. The era5 global reanalysis. *Quarterly Journal of the Royal Meteorological Society*, 146(730):1999–2049, 2020.

Set	Control	ΔT_m	ΔT_v	ΔT_{m+v}	ΔP_m	ΔP_v	ΔP_{m+v}	ΔT_m + ΔP_m	ΔT_v + ΔP_v	ΔT_{m+v} + ΔP_{m+v}
Net Ecosystem Exchange [PgC yr^{-1}]	-0.06	0.02	-0.04	0.02	-0.04	-0.04	-0.04	0.03	-0.04	0.03
Net Primary Production [PgC yr^{-1}]	1.14	1.02	1.09	0.98	1.07	1.11	1.11	0.97	1.08	0.97
Gross Primary Production [PgC yr^{-1}]	2.32	2.12	2.30	2.03	2.25	2.35	2.35	1.94	2.28	1.98
Ecosytem Respiration [PgC yr^{-1}]	2.00	1.93	2.03	1.85	1.99	2.07	2.07	1.77	2.01	1.81
Total Ecosystem Carbon [PgC]	40.36	37.28	40.45	36.96	40.59	40.81	40.81	36.86	40.44	36.85
Total Vegetation Carbon [PgC]	5.76	4.17	5.92	3.96	6.05	6.25	6.25	3.77	5.90	3.85
Total SOM Carbon [PgC]	31.72	30.78	31.75	30.69	31.83	31.79	31.79	30.82	31.78	30.70

Table S1: Carbon budgets for various scenarios. The budgets represent carbon fluxes across the entire study domain averaged over the last twenty years of the simulation.

Set	ΔT_m	ΔT_v	ΔT_{m+v}	ΔP_m	ΔP_v	ΔP_{m+v}	ΔT_m + ΔP_m	ΔT_v + ΔP_v	ΔT_{m+v} + ΔP_{m+v}
Net Primary Production	0.73	0.41	0.81	0.62	0.27	0.27	0.83	0.51	0.82
Gross Primary Production	0.78	0.13	0.82	0.46	0.21	0.21	0.87	0.22	0.84
Ecosytem Respiration	0.76	0.19	0.80	0.37	0.55	0.55	0.87	0.19	0.84
Net Rate of N Mineralization	0.95	0.04	0.95	0.12	0.02	0.02	0.94	0.07	0.95
Net Rate of P Mineralization	0.84	0.04	0.78	0.07	0.03	0.03	0.75	0.05	0.75
Total Ecosystem Carbon	0.95	0.45	0.95	0.69	0.78	0.78	0.95	0.49	0.96
Total Vegetation Carbon	0.93	0.53	0.92	0.71	0.86	0.86	0.94	0.55	0.93
Total SOM Carbon	0.95	0.46	0.95	0.65	0.54	0.54	0.95	0.52	0.95
Latent Heat Flux	0.99	0.06	0.99	0.72	0.12	0.12	0.95	0.20	0.98
Sensible Heat Flux	0.48	0.80	0.64	0.25	0.63	0.63	0.55	0.99	0.72

Table S2: The fraction of grid cells for which significant change occurred between the future set and the control set. The significance of changes is assessed by a two-sided t-test at the 95% confidence level.

Set	Control	ΔT_m	ΔT_v	ΔT_{m+v}	ΔP_m	ΔP_v	ΔP_{m+v}	ΔT_m + ΔP_m	ΔT_v + ΔP_v	ΔT_{m+v} + ΔP_{m+v}
Latent Heat Flux [W m^{-2}]	50.2	60.5	50.0	60.1	48.2	49.9	49.9	57.0	49.6	59.2
Sensible Heat Flux [W m^{-2}]	22.1	20.7	24.7	22.5	23.0	24.0	24.0	22.4	26.1	24.1

Table S3: Spatially and temporally averaged energy fluxes for various scenarios. The temporal averaging was performed over the last twenty years of the simulation.

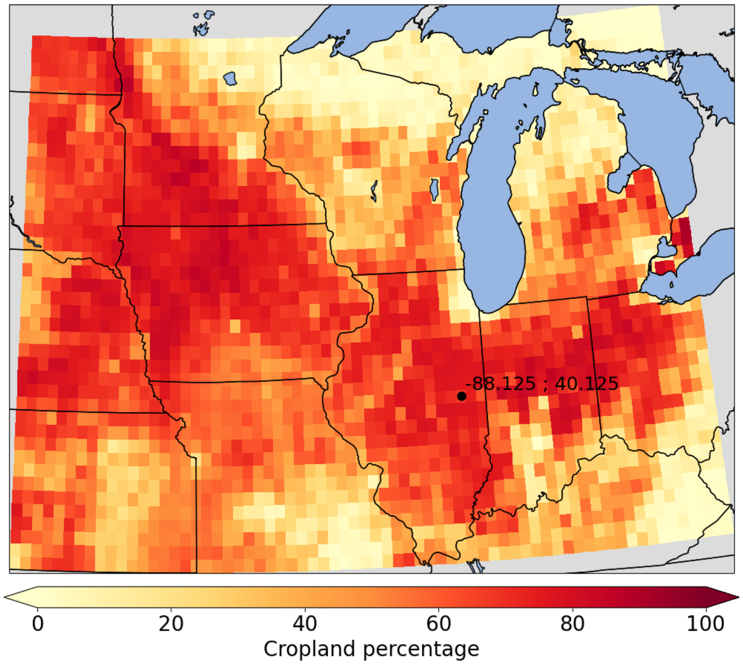

Figure S1: Study domain and percentage of cropland area in each grid cell. Also shown is the location of select grid cell in the domain for which grid cell level analysis are performed. Percent area for all grid cells are shown for year 2000.

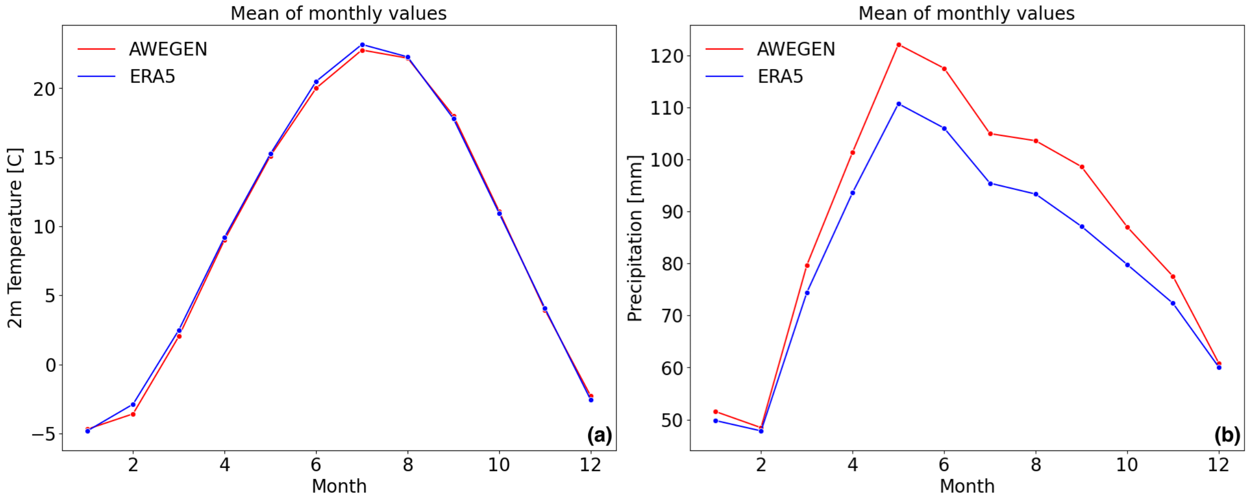

Figure S2: Observed and AWE-GEN simulated values of air temperature and precipitation. Observed (ERA5) and simulated mean monthly temperature (a) and precipitation (b) across the entire domain.

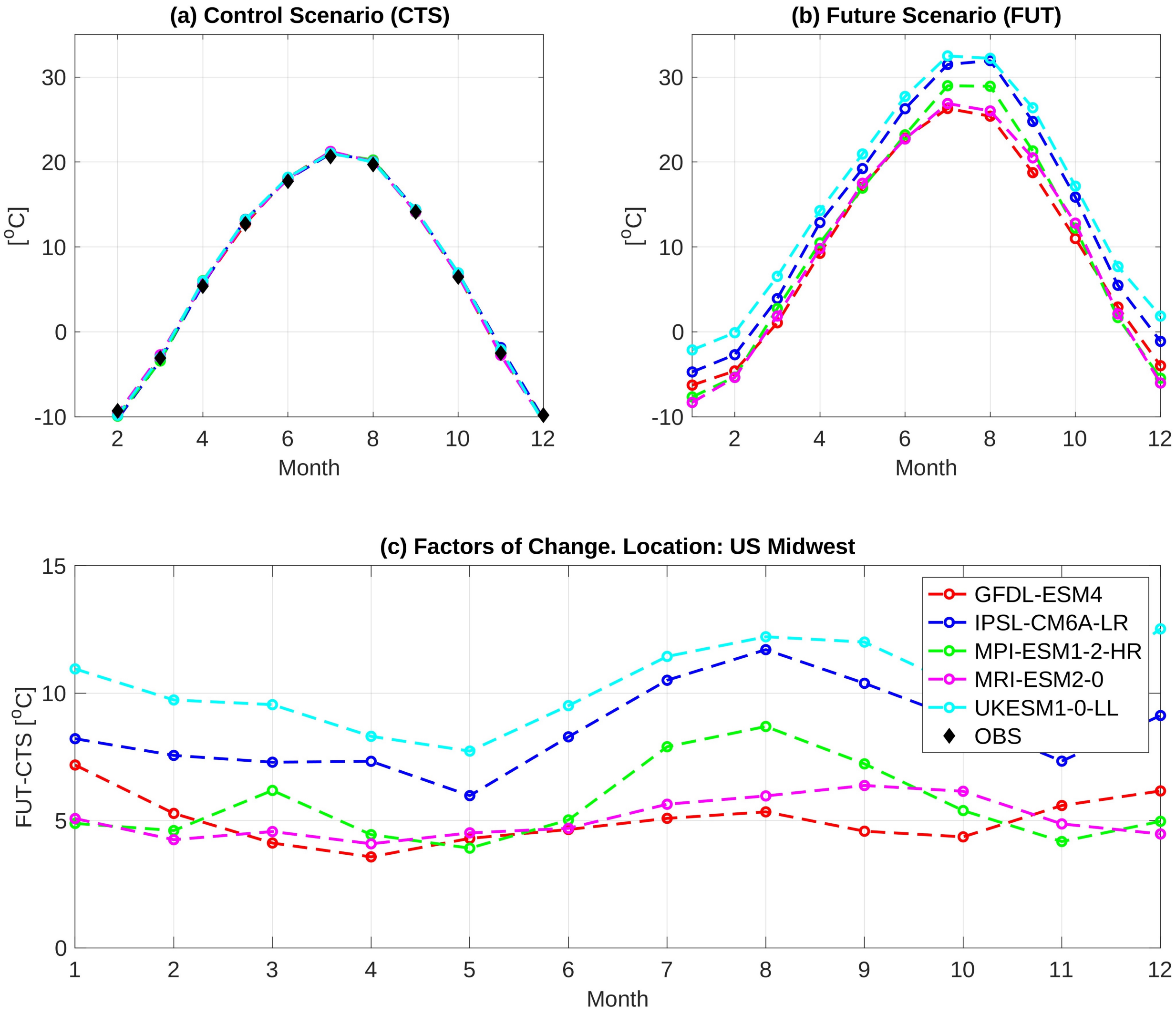

Figure S3: Mean monthly temperature for the control (a) and future scenario (b) across the entire study domain. For both scenarios temperature is based on five ESMs: GFDL-ESM4, IPSL-CM6A-LR, MPI-ESM1-2-HR, MRI-ESM2-0, and UKESM1-0-LL. The control scenario also shows observed monthly temperature based on ERA5 data. Control scenario is from 1980-2009 and future scenario from 2071-2100. Additive factors of change for mean monthly temperature (c).

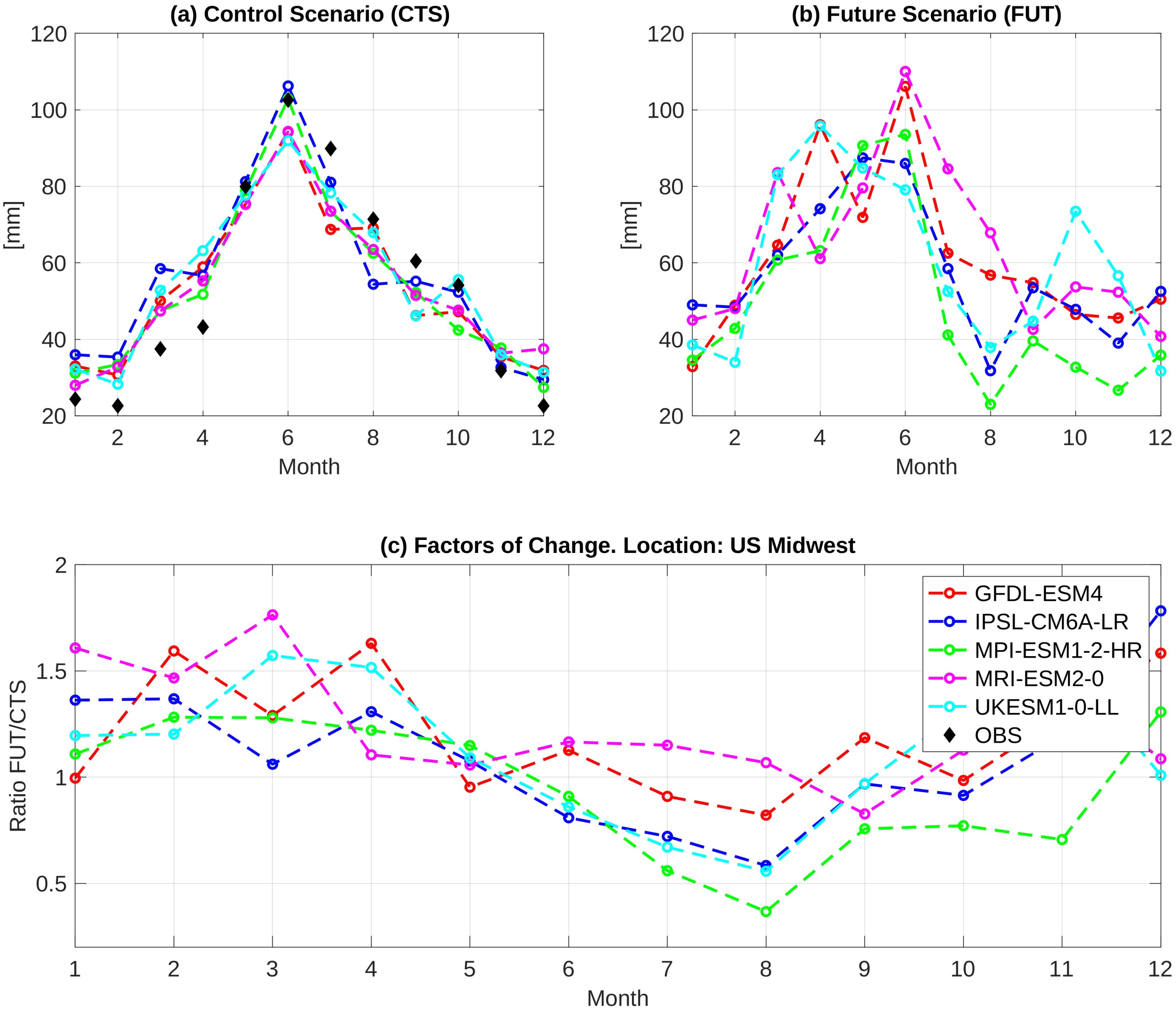

Figure S4: Mean monthly precipitation for the control (a) and future scenario (b) across the entire study domain. For both scenarios precipitation is based on five ESMs: GFDL-ESM4, IPSL-CM6A-LR, MPI-ESM1-2-HR, MRI-ESM2-0, and UKESM1-0-LL. The control scenario also shows observed monthly precipitation based on ERA5 data. Control scenario is from 1980-2009 and future scenario from 2071-2100. Multiplicative factors of change for mean monthly temperature (c).

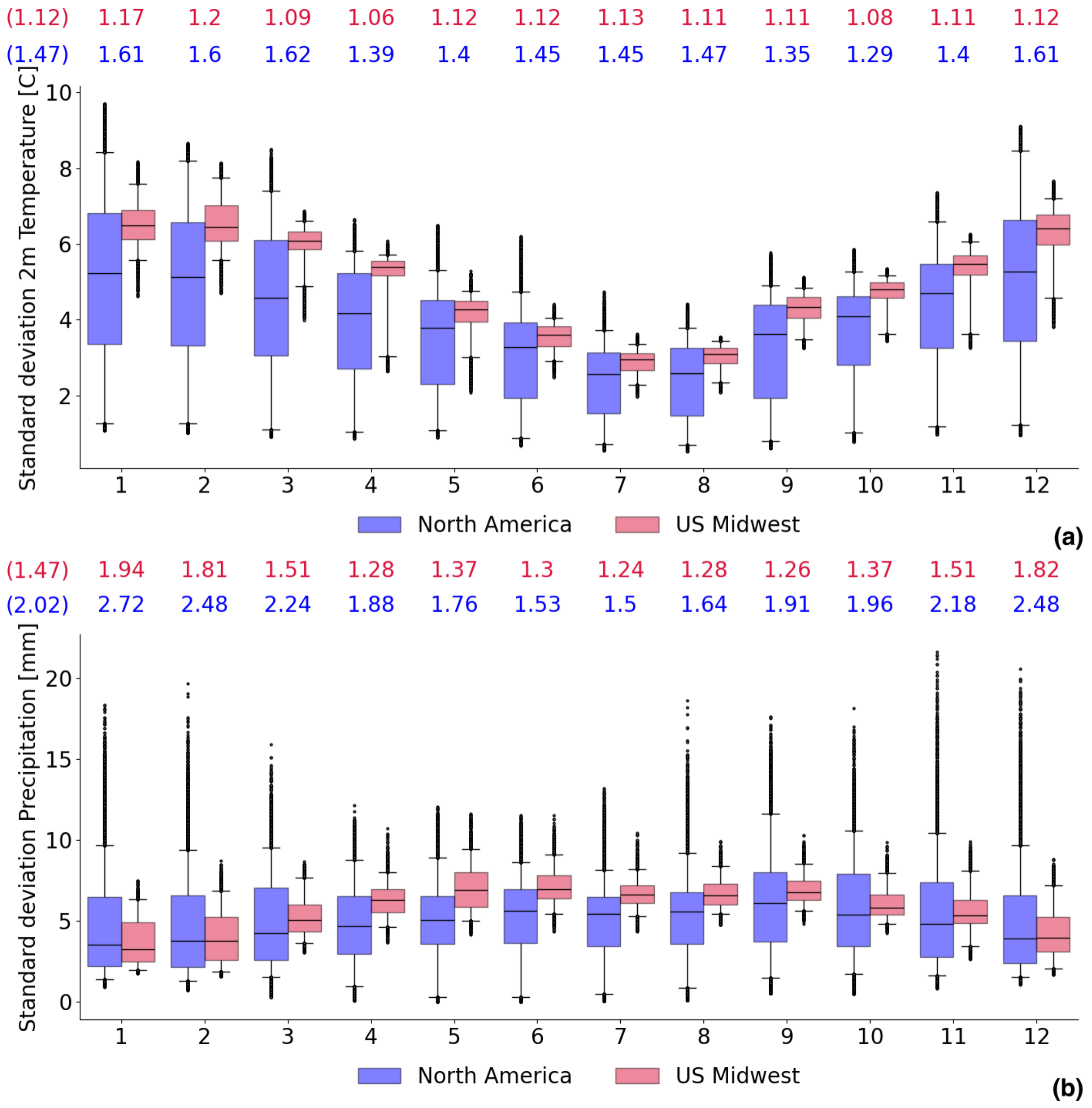

Figure S5: Spread of standard deviation of (a) daily temperature [°C] and (b) daily precipitation [mm] for each grid cell in North America (blue color box plot) and the study domain in US Midwest (crimson color box lot). Temperature and precipitation for both regions are based on ERA5 observational data [1]. The edges of the box represent 25th and 75th quantile; the line in the center of the box represents 50th quantile; and whiskers represent 5th and 95th quantile. The numbers on top represents ratio of 95th percentile to 50th percentile of the North America (blue font) and US Midwest (crimson font) data. The numbers in the parentheses on the top left is the across month average of ratio of 95th percentile to 50th percentile.

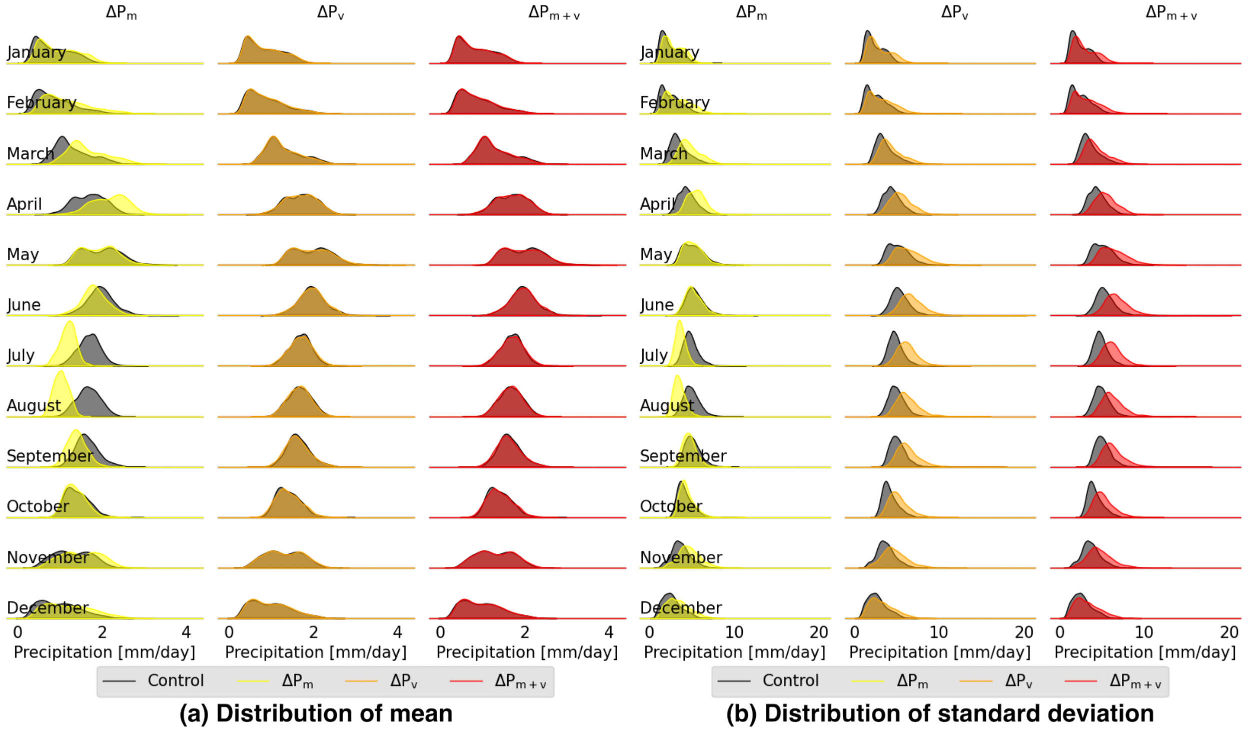

Figure S6: Modification of precipitation forcing for the future sets: Distribution of mean (a) and standard deviation (b) of daily precipitation [mm] for each grid cell.

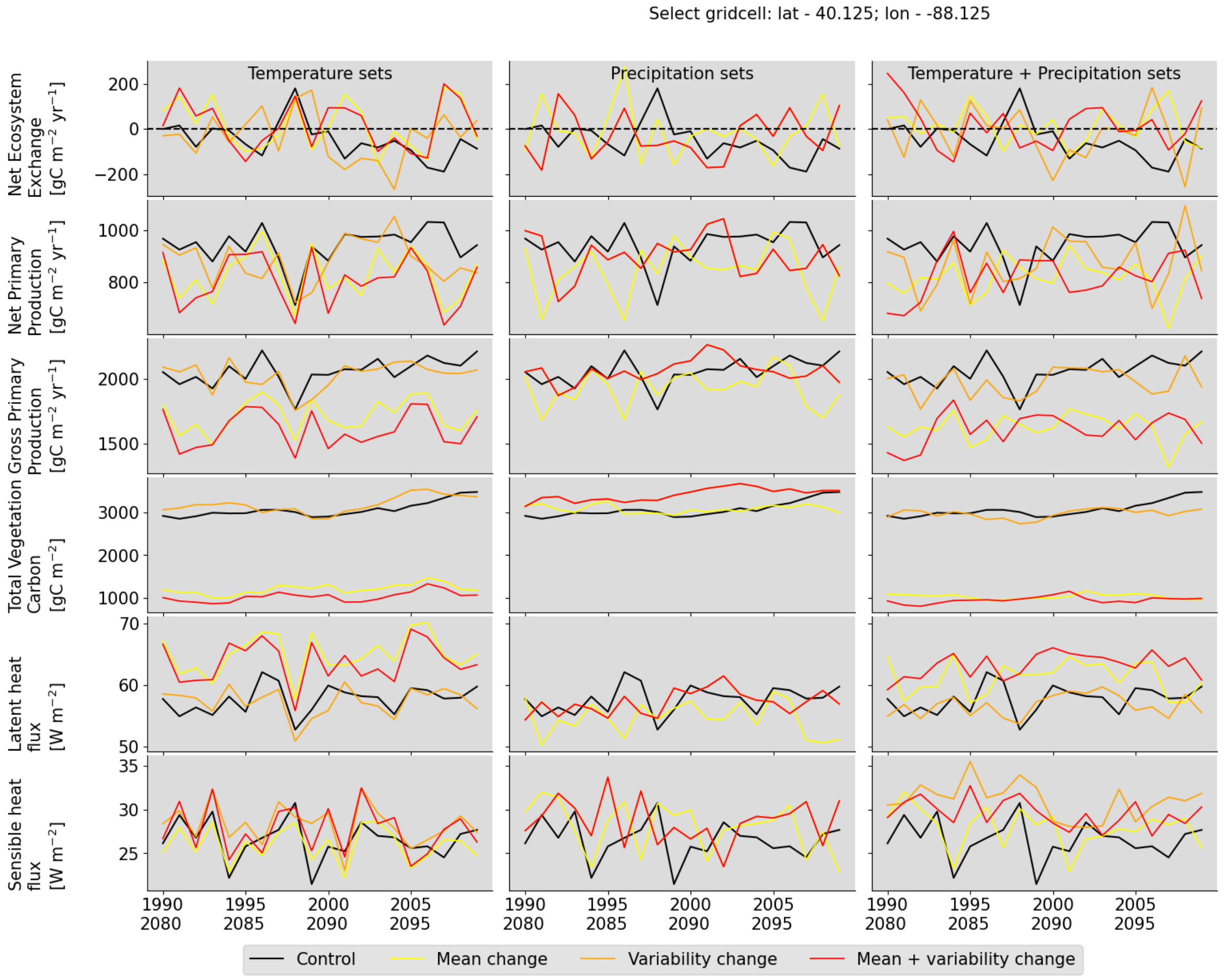

Figure S7: Time series of various carbon and energy fluxes for a single grid in the study region: control set from 1980-2009 (black color), and future sets from 2071-2100. Sets with modification to temperature mean and/or variability shown in left column (ΔT_m, ΔT_v, and ΔT_{m+v}), sets with modification to precipitation mean and/or variability shown in middle column (ΔP_m, ΔP_v, and ΔP_{m+v}), and sets with modification to both temperature and precipitation mean and/or variability shown in right column ($\Delta T_m + \Delta P_m$, $\Delta T_v + \Delta P_v$, and $\Delta T_{m+v} + \Delta P_{m+v}$). Future sets with change in temperature and/or precipitation mean shown in yellow color (ΔT_m, ΔP_m, and $\Delta T_m + \Delta P_m$). Future sets with change in temperature and/or precipitation variability shown in orange color (ΔT_v, ΔP_v, and $\Delta T_v + \Delta P_v$). Future sets with change in temperature and/or precipitation mean and/or variability shown in red color (ΔT_{m+v}, ΔP_{m+v}, and $\Delta T_{m+v} + \Delta P_{m+v}$).

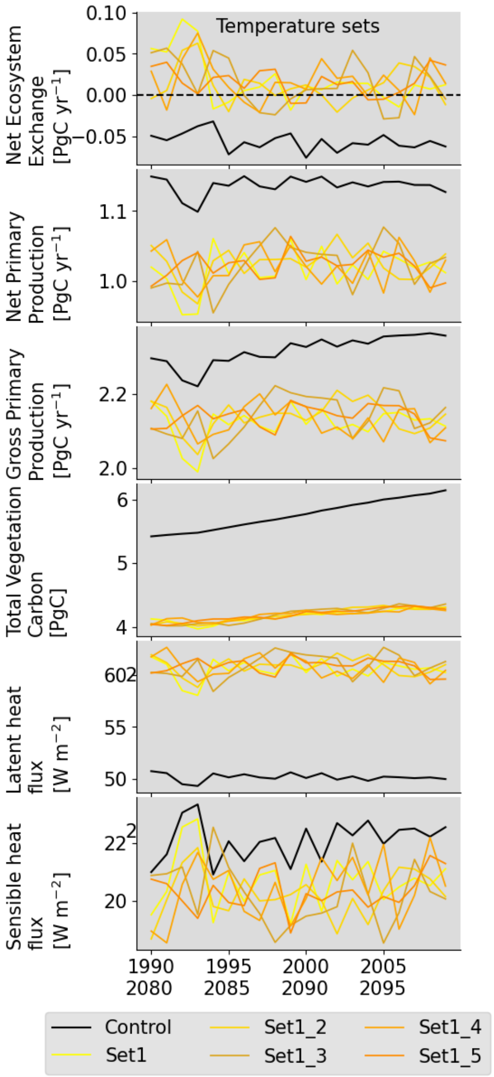

Figure S8: Time series of various carbon and energy fluxes for a single grid in the study region for: control set from 1980-2009 (red color), and future sets from 2071-2100. All future sets consider change in mean temperature but were generated with a different random number to capture the impact of internal variability.

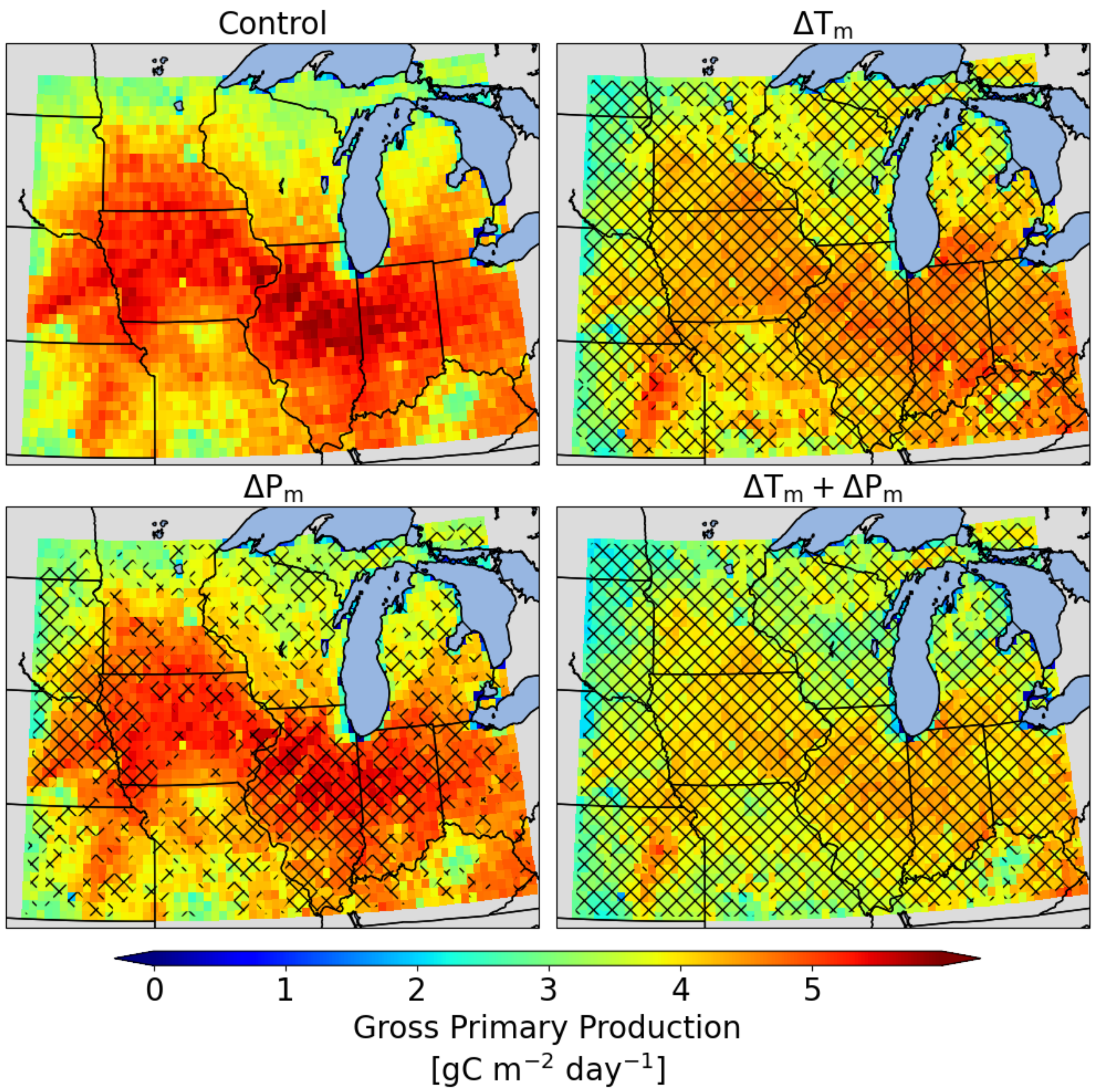

Figure S9: Gross primary productivity (GPP) for the Control, ΔT_m, ΔP_m, and $\Delta T_m + \Delta P_m$ simulations over the last 20 years of simulation. Stippling indicate significant change between control (1990-2009) and future scenarios (2080-2099).

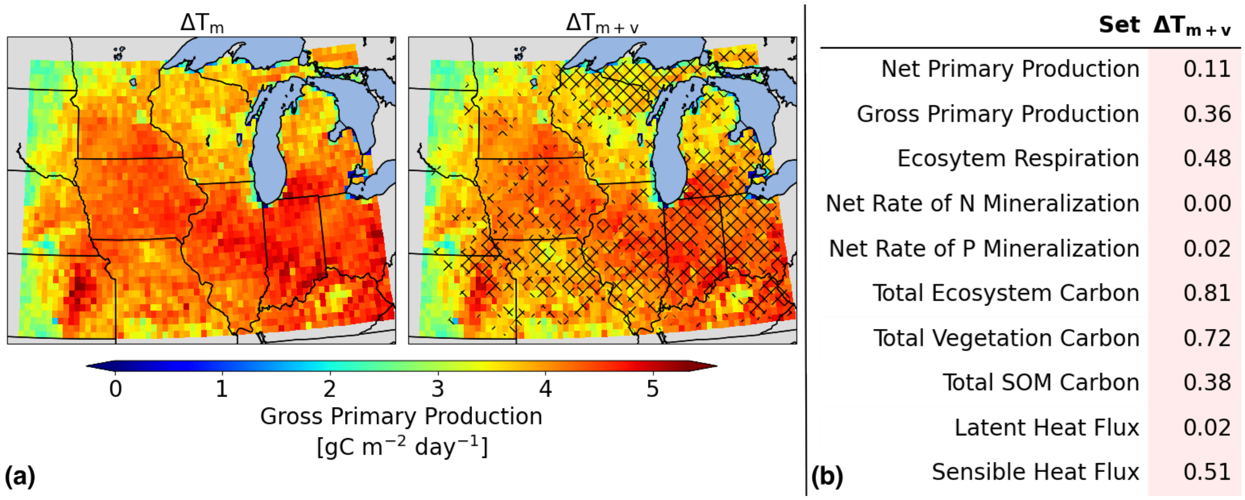

(b)

Set	ΔT_{m+v}
Net Primary Production	0.11
Gross Primary Production	0.36
Ecosytem Respiration	0.48
Net Rate of N Mineralization	0.00
Net Rate of P Mineralization	0.02
Total Ecosystem Carbon	0.81
Total Vegetation Carbon	0.72
Total SOM Carbon	0.38
Latent Heat Flux	0.02
Sensible Heat Flux	0.51

Figure S10: (a) Gross primary productivity (GPP) for the ΔT_m and ΔT_{m+v} simulations over the last 20 years of simulation. Stippling indicate significant change between ΔT_{m+v} and ΔT_m. (b) The fraction of grid cells for which significant change occurred between ΔT_{m+v} and ΔT_m.

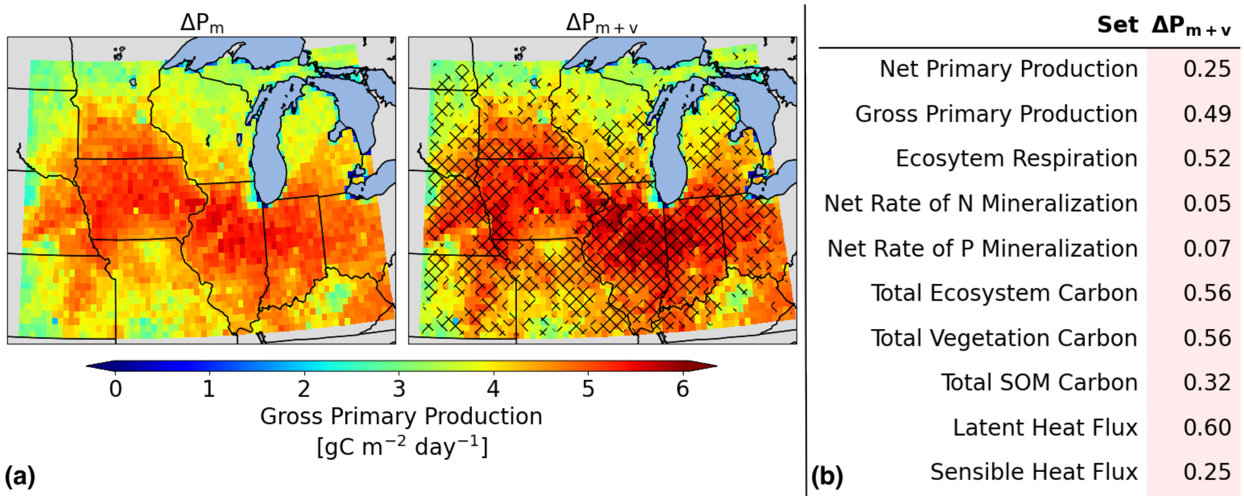

(b)

Set	ΔP_{m+v}
Net Primary Production	0.25
Gross Primary Production	0.49
Ecosytem Respiration	0.52
Net Rate of N Mineralization	0.05
Net Rate of P Mineralization	0.07
Total Ecosystem Carbon	0.56
Total Vegetation Carbon	0.56
Total SOM Carbon	0.32
Latent Heat Flux	0.60
Sensible Heat Flux	0.25

Figure S11: (a) Gross primary productivity (GPP) for the ΔP_m and ΔP_{m+v} simulations over the last 20 years of simulation. Stippling indicate significant change between ΔP_{m+v} and ΔP_m. (b) The fraction of grid cells for which significant change occurred between ΔP_{m+v} and ΔP_m.

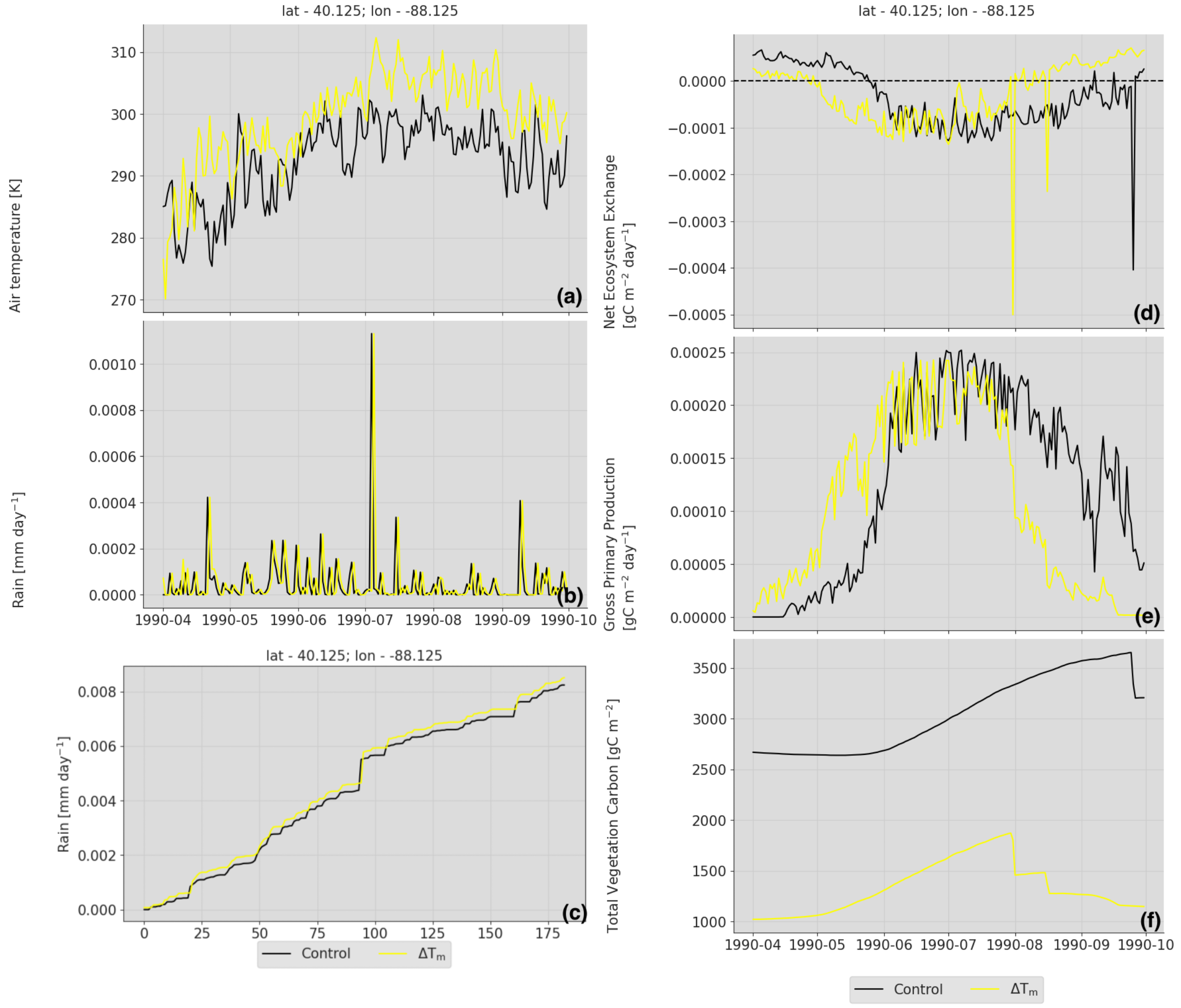

Figure S12: Grid-cell-scale impact of reduced temperature on daily carbon fluxes. Time series plots show gridcell scale daily temperature (a), daily rainfall (b), cumulative rainall (c), net ecosystem exchange (d), gross primary productivity (e), and total vegetation carbon (f).

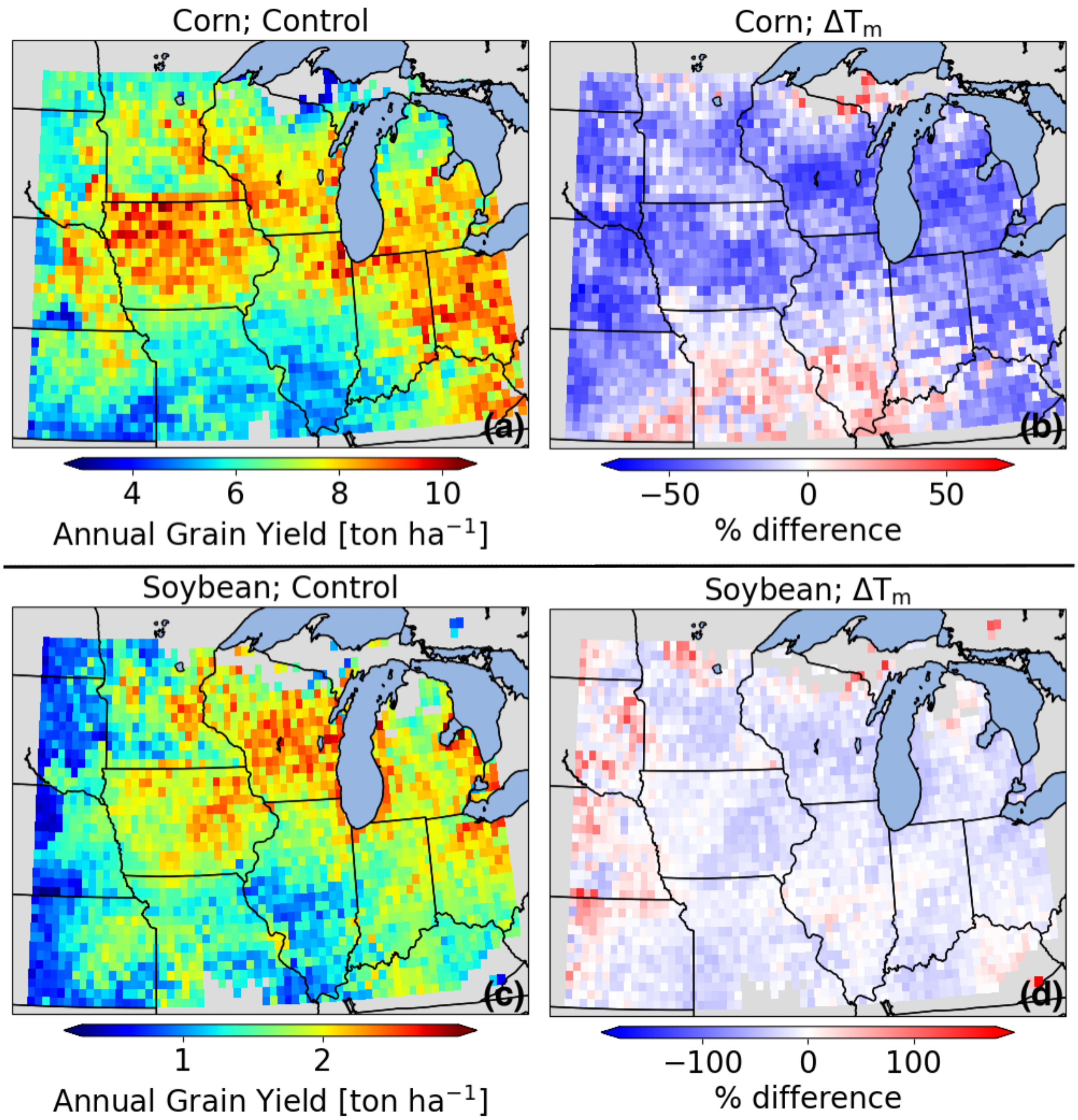

Figure S13: Annual yield for corn (a) and soybean (c) for the Control period. Percent difference in corn yield (b) and soybean yield (d) between ΔT_m and the Control set. All estimates based on the last 20 years of simulation.

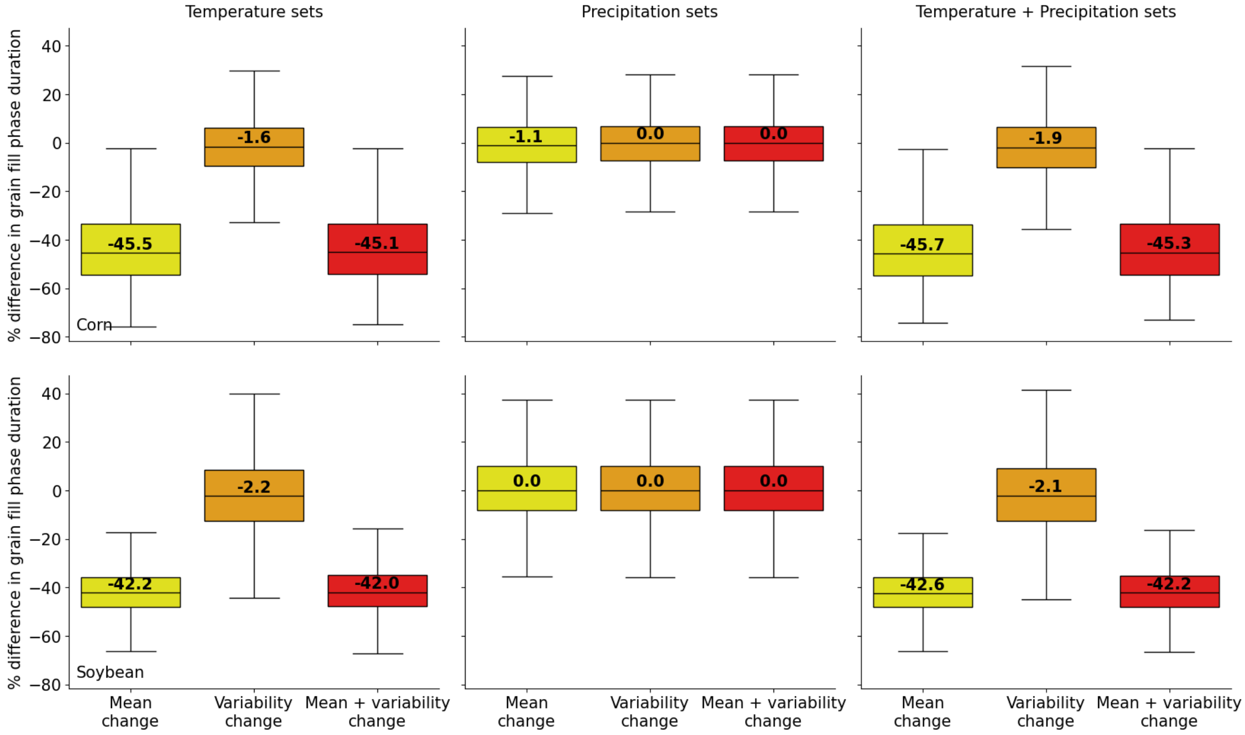

Figure S14: Distribution of percentage difference in grain fill phase duration between future sets and control set for corn and soybean. The boxplots show the distribution of percentage difference across all grid cells and over the last 20 year period. The number in the center of the boxplots represent the median value of the boxplot. The outlier points are removed to increase plot's readability.

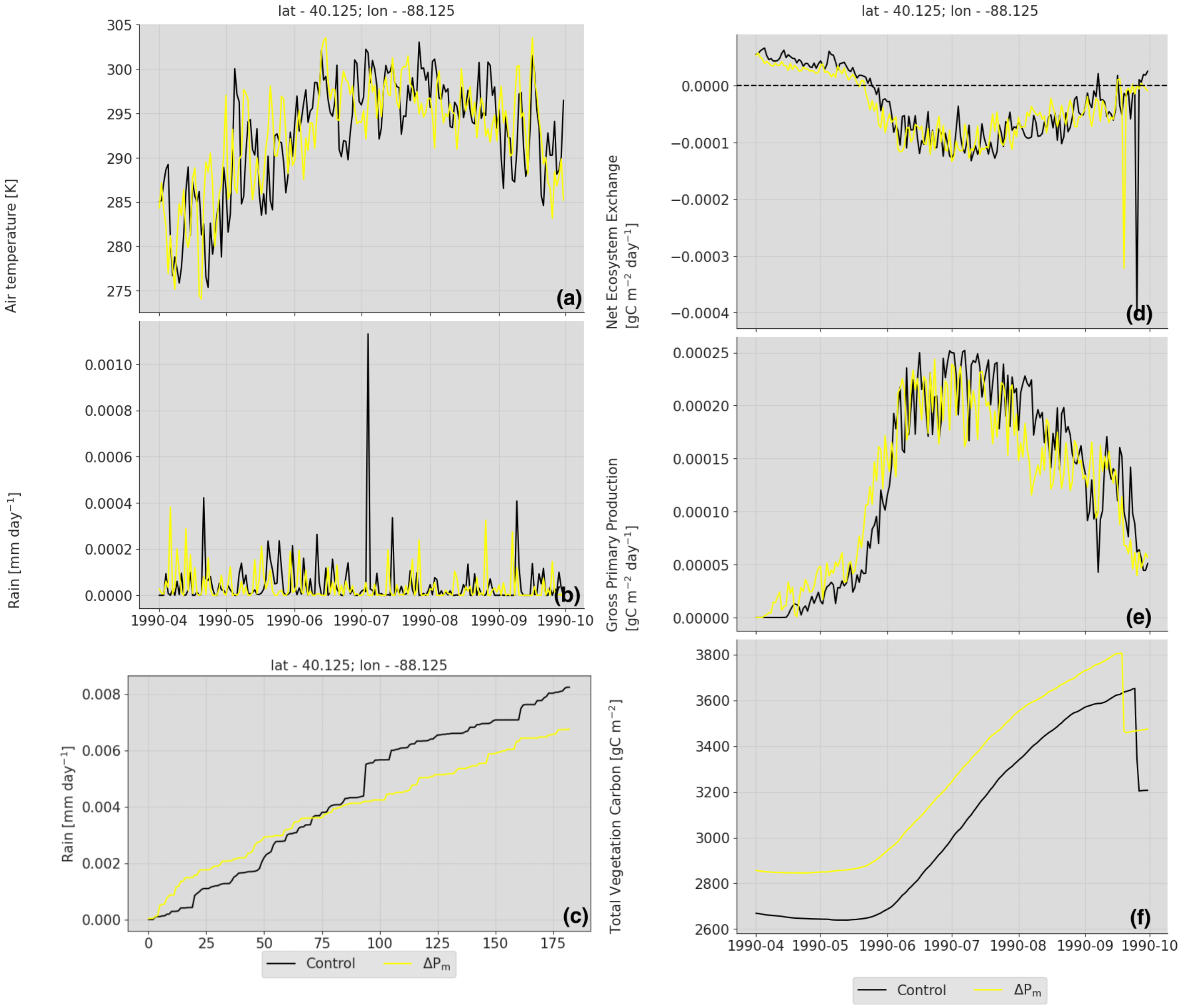

Figure S15: Grid-cell-scale impact of reduced precipitation on daily carbon fluxes. Time series plots show gridcell scale daily temperature (a), daily rainfall (b), cumulative rainall (c), net ecosystem exchange (d), gross primary productivity (e), and total vegetation carbon (f).
